# Design, synthesis, anticancer properties, and molecular docking of imidazolone derivatives with lipophilic moiety

**DOI:** 10.1038/s41598-025-97478-2

**Published:** 2025-05-27

**Authors:** Oswa Fares, Othman Hamed, Mohyeddin Assali, Avni Berisha, Haythem Saadeh, Bahia Abu Lail, Omar Dagdag, Abdullah Samaro, Waseem Mansour, Nidal Jaradat, Saber Abu-Jabal

**Affiliations:** 1 Department of Chemistry, Faculty of Science, An-Najah National University, P.O. Box 7, Nablus, Palestine; 2 Department of Pharmacy, Faculty of Medicine and Health Sciences, An-Najah National University, P.O. Box 7, Nablus, Palestine; 3 Department of Chemistry, Faculty of Natural and Mathematics Science, University of Prishtina, 10000 Prishtina, Kosovo; 4 Materials Science-Nanochemistry Research Group, Nano Alb-Unit of Albanian Nanoscience and Nanotechnology, 1000 Tirana, Albania; 5 Department of Chemistry, Faculty of Science, The University of Jordan, Amman, 11942 Jordan; 6 Department of Mechanical Engineering, Gachon University, Seongnam, 13120 Republic of Korea; 7 Department of Biomedical Sciences, Faculty of Medicine and Health Sciences, An-Najah National University, P.O. Box 7, Nablus, Palestine

**Keywords:** Vanillin, Antitumor, Lipophilic, Imidazolone, Thiophene, ADME, Cancer, Chemical biology, Chemistry

## Abstract

As part of an ongoing investigation into imidazolone derivatives with anticancer activities, herein we present the synthesis of a new series of imidazolones with various substituents, including lipophilic and hydrophilic. All synthesized imidazolones (3a–3g and 5a–5g) were characterized by various spectroscopic methods (^1^H and ^13^C NMR, FT-IR, and mass spectrometry). The preparation was performed by condensation cyclization of vanillin-based oxazolones with various amines. The anticancer efficiencies of the prepared imidazolones were tested against four different cancer cell lines liver cancer cells (HepG2), cervical adenocarcinoma cells (HeLa), colon cancer cells (CaCo-2), breast cancer cells (MCF-7). Among the prepared imidazolones the one with dodecyl chain displayed excellent efficiency against the tested cancer cell lines with an IC_50_ value of 65.3 ± 3.2 µM against HepG2 and 20.02 ± 3.5 µM against MCF-7. Imidazolone 2d with amino alkyl moiety displayed an IC_50_ value of 35.6 ± 4.1 µM against HeLa cell and 24.6 ± 3.8 µM against CaCo-2 cell line. Imidazolone 5g with thiophene and pyridyl group showed the highest activity among all tested derivatives with IC_50_ value of 18.6 ± 2.3 µM and 5.9 ± 2.3 µM against HeLa and CaCo-2 cell lines, respectively. Imidazolone 5b with a chlorophenyl moiety displayed an IC_50_ value of 2.2 ± 0.7 µM and 5.5 ± 1.1 µM against HepG2 and Hela cell lines, respectively. The study used computational tools to assess the pharmacokinetics and antitumor potential of the synthesized imidazolone molecules with the highest activities. They were evaluated through ADME analysis and molecular docking. ADME properties confirm favorable drug-likeness under Lipinski’s guidelines, with molecular weights ranging from 357.43 (5d) to 468.65 g/mol (5f). Molecules 2g, 2f, and 5f show optimal hydrogen bonding, moderate bioavailability (0.55), and synthetic accessibility scores from 3.78 to 4.76. Docking studies with proteins 4MAN and 1HNJ highlight strong interactions for 2g, 2f, and 5f, with molecule 3g showing the best binding for 4MAN (− 52.13 kcal/mol) and 5f for 1HNJ (− 38.63 kcal/mol). These results identify 3g and 5f as promising candidates for targeted cancer therapy.

**Supplementary Information:**

The online version contains supplementary material available at 10.1038/s41598-025-97478-2.

## Introduction

The creation of novel, selective anticancer agents has garnered significant attention from chemists due to the high death rate associated with current cancer drugs^1^. Since commercially available anticancer medications have a wide range of adverse effects, poor selectivity, toxicity, and drug resistance. Finding novel, potent, and selective anticancer medications with limited adverse effects is therefore crucial^2,3^. Heterocyclic compounds, especially those that contain nitrogen atom(s), hold a crucial place in the toolbox of contemporary medicinal and organic chemistry due to their wide range of physiological activity^4^. Furthermore, a variety of pharmacologically active compounds and natural products have one or more heterocyclic compounds in their structure^5^, including enzymes, protein, nucleic acids, and vitamins. All of these contain nitrogen frameworks are necessary for a living thing to operate. Therefore, heterocyclic-based compounds could be crucial in the development of new bioactive substances with anticancer activity.

Within the realm of the heterocyclic derivatives, imidazolones and imidazoles^6–10^ have a variety of biological and pharmacological potentials^11–13^, such as anti-convulsant^14,15^, cardiovascular, anti-tubercular, anti-bacterial, and monoamine oxidase (MAO) inhibitory effects^16,17^. Moreover, some imidazolone and imidazole derivatives showed more selective bioactivities such as anti-muscarinic, anti-inflammatory, anticancer and anti-histamine^18^ (1). More detailed information about their method of making and bioactivities is presented in a review by Tolomeu et al.^19^. Imidazoles also forms the fundamental building blocks of several naturally occurring heterocycles, including purines, histidine, histamines, and DNA^20^.

Several chemotherapeutic drugs known for their high potency, like Etoposide, Methotrexate, and Paclitaxel exhibit major drawbacks, including high toxicity, severe side effect, and drug resistance. According to some published research, imidazoles may be able to get beyond these challenges and show promise as anticancer medicine with a variety of possible modes of action^20-22^.

**Fig. 1:**
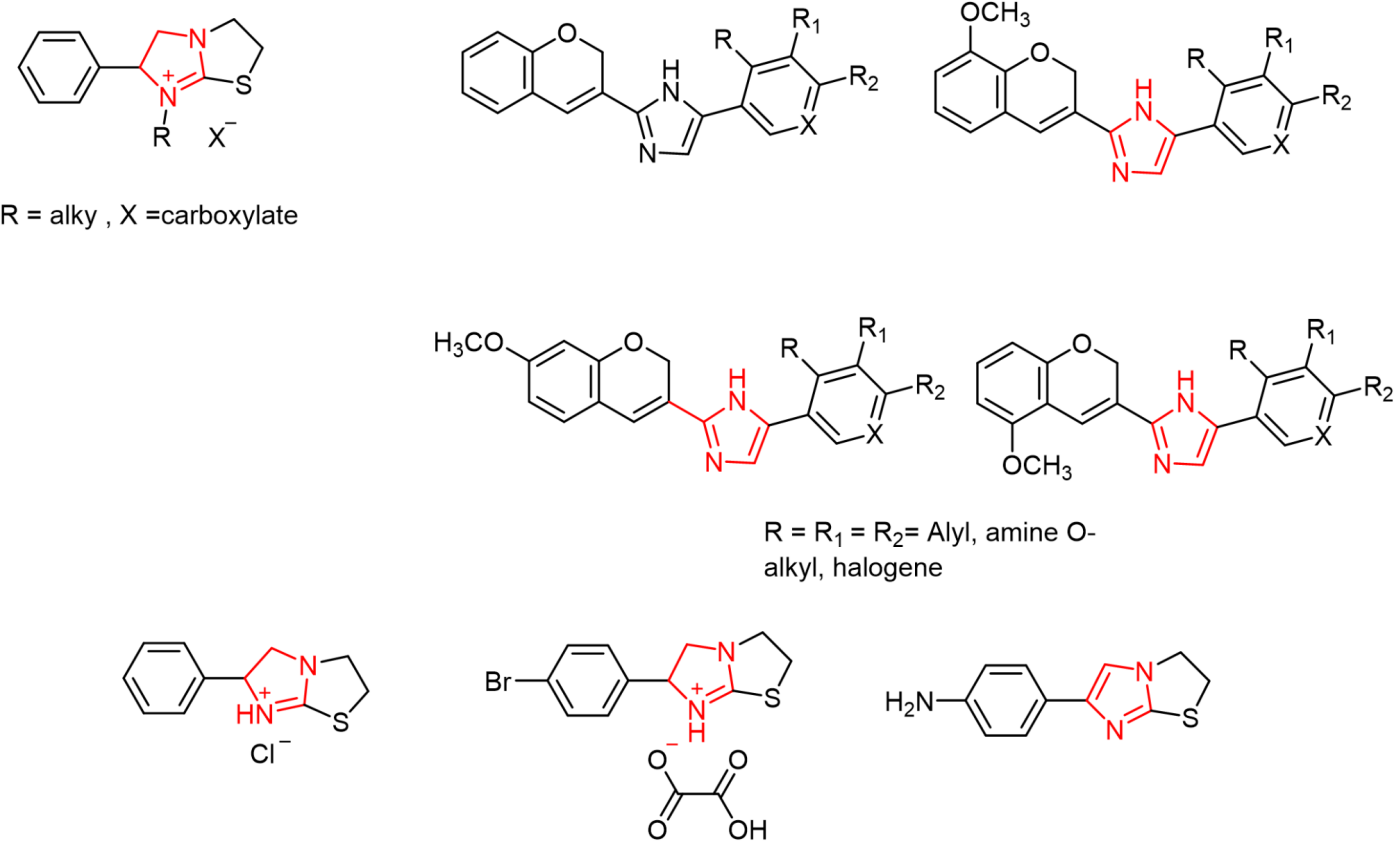
Imidazoles with antiangiogenesis activity.

**Fig. 2:**
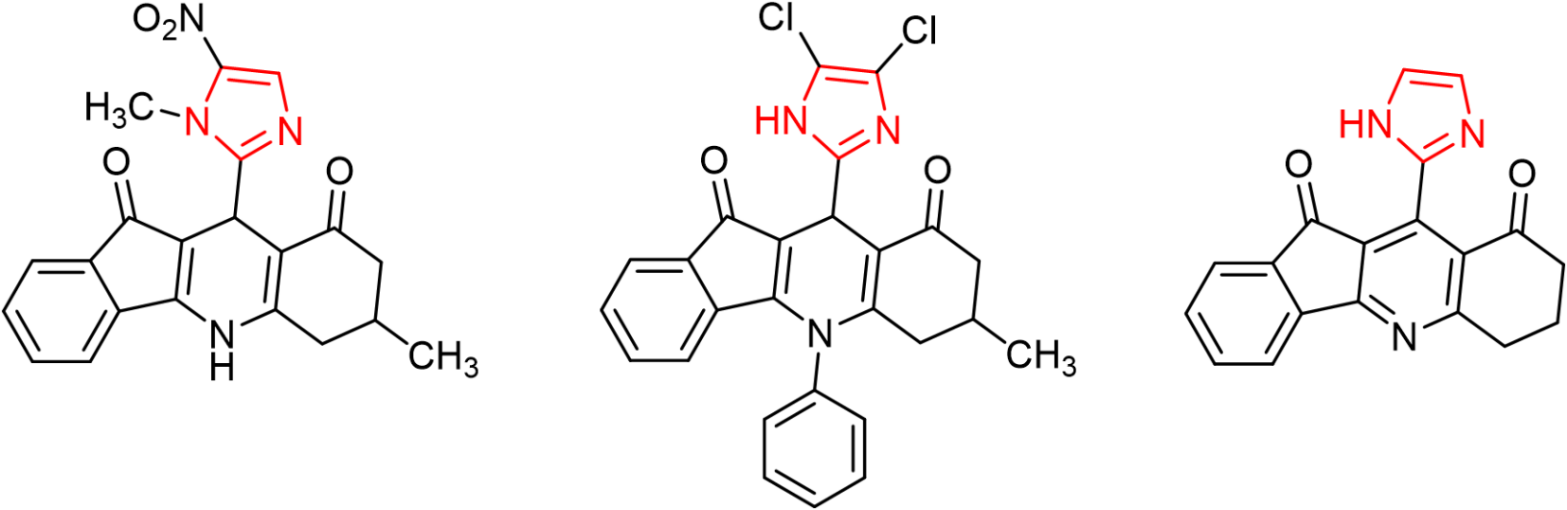
Imidazoles with antiproliferative activity.

Imidazoles with various functionalities were designed as anticancer agents, some of them showed the ability to overcome the various drawbacks of currently available anticancer drugs. This was attributed to their various mechanisms of action via various targets like DNA, histone deacetylases, VEGF, receptor tyrosine kinases, mitotic spindle microtubules, CYP26A1 enzyme, topoisomerases, and rapid accelerated fibrosarcoma (RAF) kinases. For instance, some imidazoles have been explored as anticancer agents with antiangiogenesis as the mode of action, they showed in vitro inhibition of angiogenesis. Examples of these are shown in Fig. 1^23^. Others like those reported by Chen et al. (Fig. 2), act as antiproliferative agents for melanoma^24^. For more detailed information on imidazole and their various modes of action against cancer one can return to a review by Ali et al. and Kumar et al.^25,26^

**Fig. 3:**
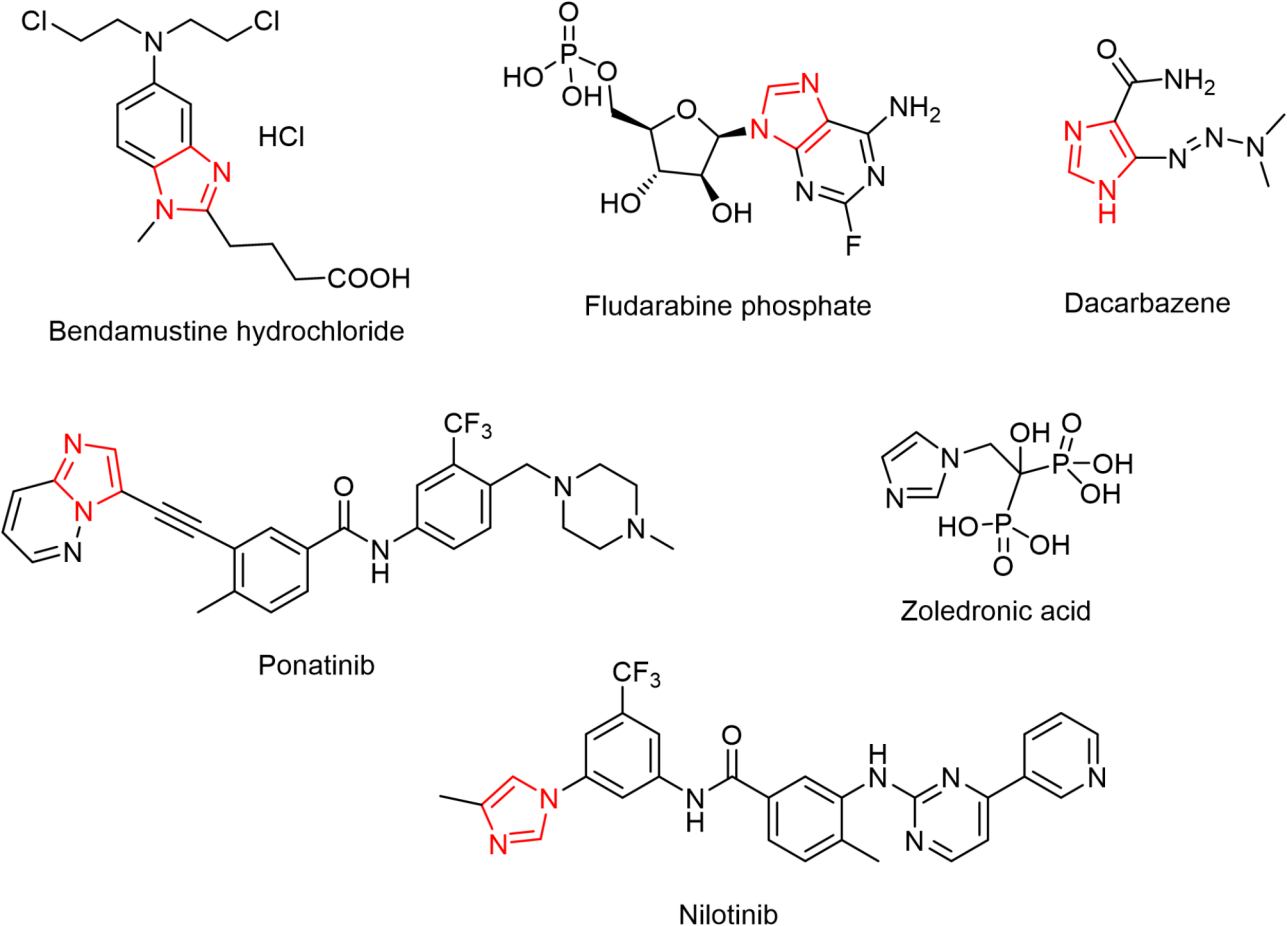
Anticancer drugs with imidazole or imidazoles fused rings.

Several imidazole derivatives have already been approved and employed as potent anticancer agents like those shown in Fig. 3. They are used to treat different types of cancer**.** In summary, many compounds containing imidazole possess exceptional pharmacological abilities to manage cancer (24).

Various synthetic methods of imidazoles are covered in several review articles. Some of these methods require improvements since they suffer from major drawbacks, like they require long reaction times, severe reaction conditions, and low yield(4). Another disadvantage is they show limited bioactivities and bioavailability due to low water solubility^27–33^.

Creating a new heterocyclic drug based on imidazolone with a variety of substituents could be a reliable approach to solving some of the mentioned issues^34^. Adding a lipophilic group to imidazolone could be a novel approach for enhancing the activity against cancer cells.

To discover new anticancer candidates with higher potency, less toxic and more selective, we report in this work the synthesis and the anticancer activities of vanillin-based imidazolones with various functionalities like a thiophane ring and lipophilic groups. To the best of our knowledge, we report herein the first example of imidazolone with lipophilic chain with excellent anticancer activities. The effectiveness of the synthesized imidazolone derivatives as selective and potent anticancer agents was examined against the liver cancer cells (HepG2), cervical adenocarcinoma cells (HeLa), colon cancer cells (CaCo-2), and breast cancer cells (MCF-7). The pharmacokinetics and antitumor potential of some of the imidazolone molecules with the highest activities were evaluated through ADME analysis and molecular docking.

The diagram shown below depicts the reasoning for structural alterations in target imidazolone derivatives, as well as the logical sequence from original design to final synthesis and biological evaluation.


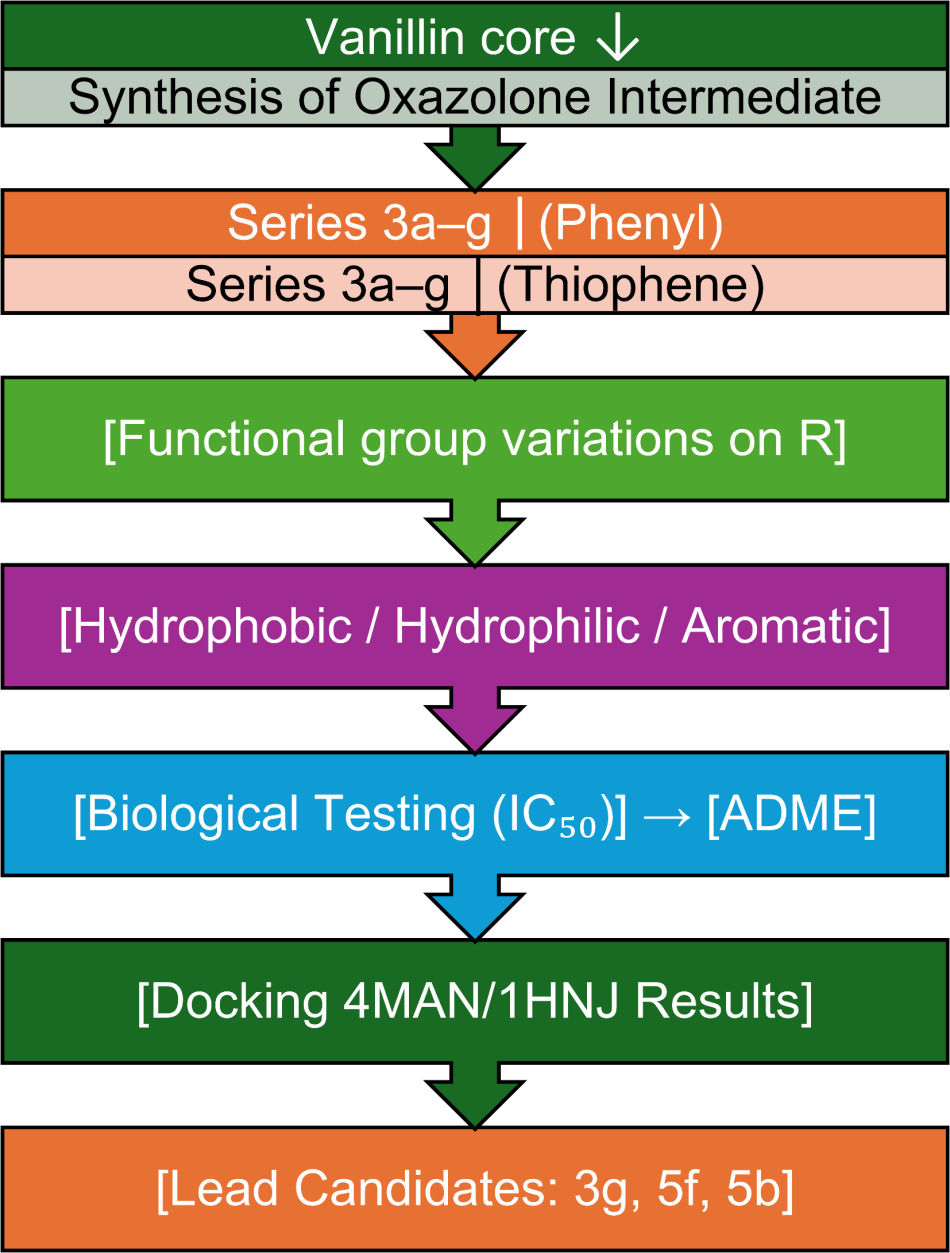


## Results and discussion

### Synthesis of imidazolone derivatives

**Scheme 1:**
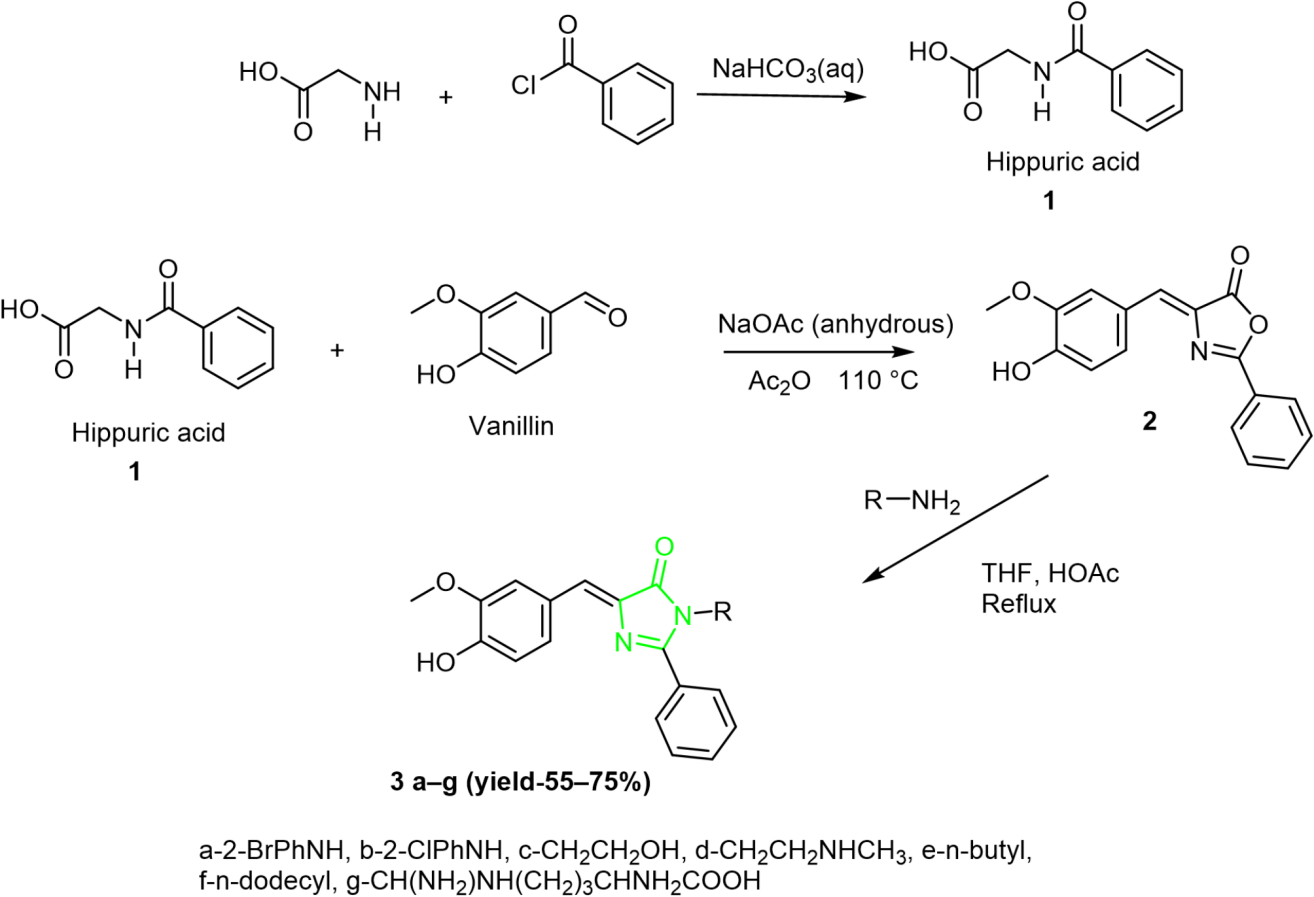
Synthesis of imidazolones **3a**–**g**.

The synthetic method used to prepare imidazolones **3a–g** is outlined in Scheme 1. The synthetic route involved a three-step process with a yield ranging from 61 to 76%. The first step involves the synthesis of hippuric acid from reacting benzoyl chloride with glycine. The Erlenmeyer-Plöchl nucleophilic substitution afforded oxazolone **2**. The reaction was carried out in the presence of sodium acetate anhydrous and acetic anhydride to remove water which produced as a side product(6). The formation of oxazolone **2** occurs through condensation and cyclization that involves the loss of water molecules. Reacting oxazolone **2** with different hydrazines and primary amines afforded the target imidazolones (**3a to f**). The amine compounds function as nucleophiles that make a nucleophilic attack on the carbonyl group of oxazolone causing a ring opening, followed by condensation, dehydration, and ring closure. Among the prepared imidazolone are those with the lipophilic groups dodecyl and butyl.

The structures of oxazolone and imidazolones (**3a to g**) were confirmed by ^1^H and C-13 NMR and MS/MS. The mass spectrum imidazolones with halogens showed the isotopic profile that indicates their presence as shown in the experimental section.

**Scheme 2:**
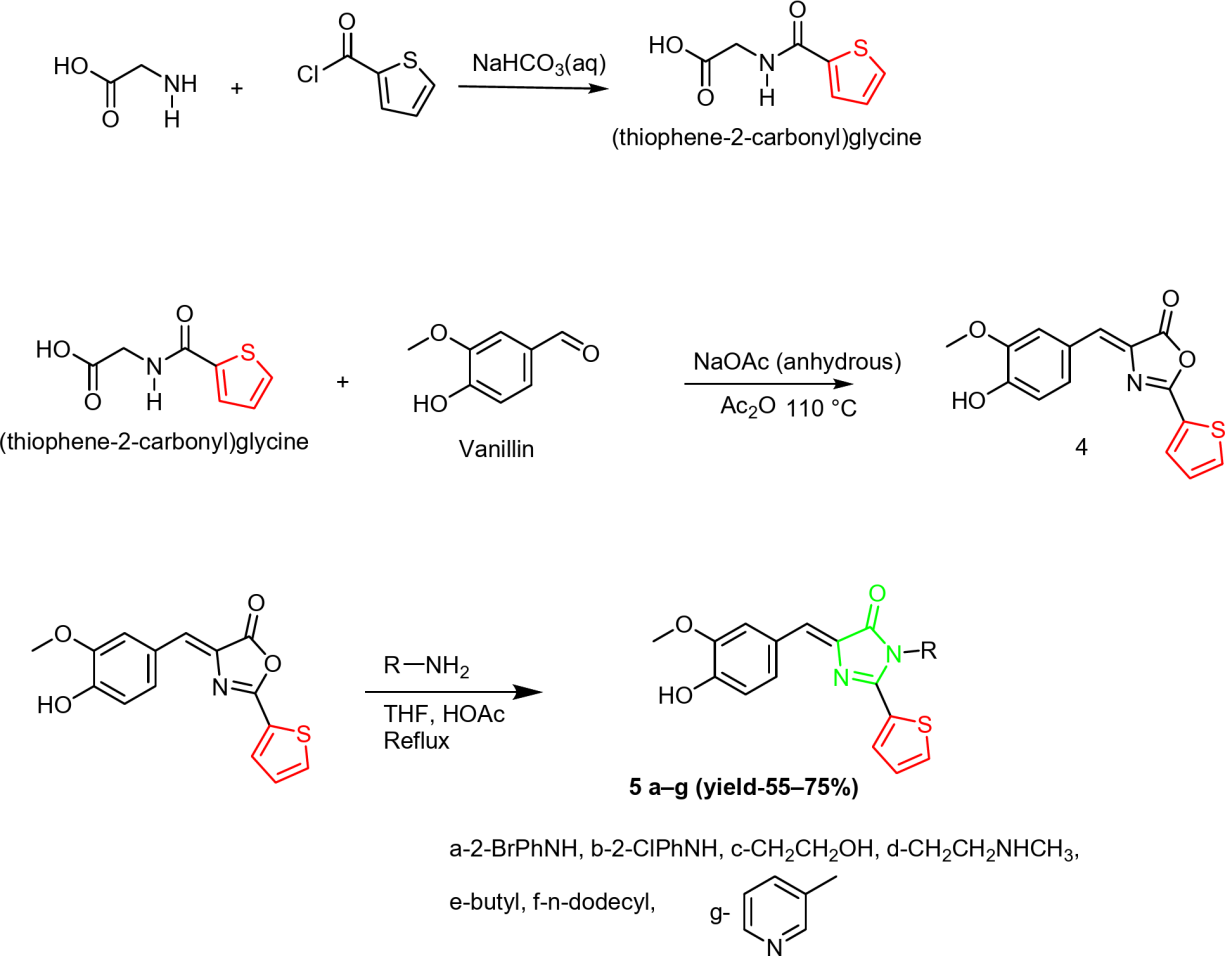
Synthesis of imidazolones **5a**–**g**.

A second set of imidazolones (**5a to g**) was prepared using the above synthesis strategy that is summarized in Scheme 2. The phenyl ring in imidazolones **3** was replaced with a thiophen ring. The heterocyclic thiophene was added to increase the binding sites in the imidazolones, as thiophene is known to have various bioactivities^35^.

The synthesis of compounds **5a–g** involved reacting 2-thiophene carbonyl chloride with glycine in the first step to produce the building block (thiophene-2-carbonyl) glycine, which then reacted with vanillin to produce oxazolone (**4**) with a thiophen ring. The reaction again was carried out in the presence of sodium acetate anhydrous and acetic anhydride**.** Reacting oxazolone **4** with different primary amines and hydrazines, including amines with lipophilic chains produced the target imidazolones **5a to g** (Scheme 2).

The structures of imidazolones (**5a to g**) were confirmed by ^1^H and C-13 NMR and MS/MS.

### Anticancer activities of imidazolones 3a–g

The anticancer screening of the prepared imidazolones against various cancer cells, including HepG2, HeLa, CaCo-2, and MCF-7 was performed by testing the viability of the cells using the MTS assay. The measured IC_50_ values for the tested compounds **3a–g** are listed in Table 1. The screening was carried out using four doses of imidazolones with various concentrations ranging from 500.0 to 62.5 μM, the solutions were prepared by dilution form a stock solution with a 1000 μM concentration. The incubation was performed for 48 h. All screening tests were performed in triplicate, the average value was reported. The IC_50_ value was determined for each cancer cell, the value represents the molar concentration of imidazolones required to kill 50% of cancer cells.

**Table 1:** IC_50_ (µM) of imidazolones **3a–g** against the cancer cells HepG2, Hela, CaCo-2, and MCF-7.

Imidazolone	IC_50_ (µM)			
	HepG2	HeLa	CaCo-2	MCF-7
3a	1484.7283	292.4388	57.9116	115.2819
3b	163.4581	151.3361	21.0425	98.4083
3c	175.4095	768.7001	50.833	427.2536
3d	210.9313	36.575	24.6784	178.3757
3e	449.5746	125.362	109.753	574.1845
3f	65.2608	111.8522	96.8992	20.02
3g	461.8246	251.7931	261.7514	715.2675

Table 1 shows the IC50 data of the various imidazolone derivatives on the four tested cancer cell lines. It was observed that compounds 3a and 3b have selective activity on colorectal cancers (CaCo-2 cells) with IC_50_ 57.9 and 21.04 µM, respectively. These two compounds have almost similar substitutes for R with Br and Cl on the aryl ring. However, compound 3b with the 2-chloro phenyl amine group showed a higher potency in comparison to the bromo derivative. Compound 2c with ethanol amine also showed selective activity on the CaCo-2 cells with IC_50_ equal to 50.8 µM. Compound 3d has an N-methyl ethylene diamine group exhibiting anticancer activity on both the colorectal cancer cells and cervical cancer cells with IC_50_ equal to 36.57 and 23.68 µM, respectively. The most potent imidazolone of all tested cancer cells was 3f with a dodecyl group, and the highest activity was on the breast cancer cells (MCF-7) with IC_50_ 20.02 µM. This could be attributed to the hydrophobic nature of the dodecyl group that could enhance the cancer cell membrane penetration. Compound 3e showed moderate activity on HeLa and CaCo-2 cells. However, compound 3g showed medium anticancer activity on HeLa and CaCo-2 cells. This could contribute to the high hydrophilic nature of the substitution, which could reduce the possibility of cancer cell penetration.

### Anticancer activities of imidazolones 5a–g

The anticancer activities of the imidazolones 5a–g were evaluated in the same manner as above on the same cancer cells lines (HepG2, HeLa, CaCo-2, and MCF-7). The IC_50_ values were determined and listed in Table 2. This class of compounds showed improved anticancer activities in comparison with the first set. This could contribute to the presence of the thiophene ring, which adds extra interaction as mentioned above. Compound 5b with a chlorophenyl moiety showed the most potent anticancer activity on the HepG2 and HeLa cancer cells with a high selectivity as they showed a very low IC_50_ of 2.18 and 5.51 µM, respectively. Compound 5a with a bromophenyl substituent exhibits good anticancer activity against the HepG2 and HeLa cancer cells with IC_50_ equal to 22.39 and 53.64 µM, respectively. While compounds 5c with ethyl alcohol substitution and 5d with N-methyl ethylene amine demonstrated selective activities on CaCo-2 cells with potent IC_50_ values of 13.171 µM for 5c and 32.7 µM for compound 5d. In the case of the butyl substitution in compound 5e, the compound displaced anticancer activity on HeLa and MCF-7 cancer cells with IC_50_ 45.7 µM and 85.8 µM, respectively. Regarding the dodecyl moiety, the compound showed moderate activity on all tested cancer cells, ranging from 67.67–114.15 µM, which confirms the capability of the hydrophobic group to increase cell penetration. Finally, compound 5g with the picoloyl moiety displayed very potent activity on the colorectal cancer cells and cervical cancer cells with IC_50_ equal to 5.96 µM and 18.44 µM, respectively. However, it has moderate activity on the liver cancer cells with IC_50_ 53.83 µM.

**Table 2:** IC_50_ (µM) of imidazolones **5a–g** against the cancer cells HepG2, HeLa, CaCo-2, and MCF-7.

Imidazolone	HepG2	HeLa	CaCo-2	MCF-7
5a	22.3947	53.6378	117.2313	162.7124
5b	2.1829	5.5135	1345.3248	191.8282
5c	239.9133	97.6824	13.171	184.3424
5d	103.0476	125.2851	32.7427	35.5975
5e	1636.66	45.7111	562.7565	85.8287
5f	67.6734	78.0116	68.4498	114.15157
5g	53.8275	18.4382	5.9562	1316.489

We could conclude that depending on the substituent of the imidazolone derivatives the anticancer activity varies on the cancer cells as well as the potency of the compounds.

The cytotoxicity of the compounds (**5a to g**) against the tested cancer is shown in Fig. 3 below.

### ADME

ADME (Absorption, Distribution, Metabolism, and Excretion) properties for the molecules were computed via the use of Swiss ADME server^36^. The most important properties for the studied molecules are presented in Table 3. The molecular weights of the molecules range from 357.43 (5d) to 468.65 (5f). According to Lipinski’s guidelines, MW ≤ 500 is optimal for drug-like molecules. All molecules fall within this range, making them potentially suitable for oral bioavailability^37^. The number of H-bond acceptors varies from 4 (3f, 5f) to 9 (3g), while H-bond donors range from 1 (3f, 5f) to 5 (3g). Molecule 3g has the highest potential for hydrogen bonding, which might improve solubility but could affect permeability. Molecule 3f has one violation, whereas the other molecules comply fully with Lipinski’s rules, indicating a higher likelihood of favorable pharmacokinetics for molecules 3g, 5f, and 5d^38,39^. All molecules share a bioavailability score of 0.55, which suggests moderate oral bioavailability. This is consistent across the dataset and aligns with the structural features of the compounds. Each molecule triggers at least one PAINS alert^40^, highlighting potential suboptimal pharmacological properties.

**Table 3:** The ADME properties for selected synthesized molecules.

Molecule	MW	#H-bond acceptors	#H-bond donors	Lipinski #violations	Bioavailability Score	PAINS #alerts	Brenk #alerts	Synthetic accessibility
3f	462.62	4	1	1	0.55	1	1	4.49
3g	453.49	9	5	0	0.55	1	2	4.76
5f	468.65	4	1	0	0.55	1	1	4.61
5d	357.43	5	2	0	0.55	1	1	3.78

Molecule 3g has two Brenk alerts, suggesting a slightly higher risk of undesirable effects compared to others. The synthetic accessibility score ranges from 3.78 (5d) to 4.76 (3g). Lower scores indicate simpler synthesis. Molecule 5d is the easiest to synthesize, whereas 3g may present challenges due to its complex structure. In general, QSAR parameters indicate that all molecules exhibit reasonable drug-like properties, with variations in hydrogen bonding and synthetic complexity that may influence their development potential. Molecule 5d emerges as the most balanced candidate due to its compliance with Lipinski’s rules, moderate H-bond properties, and favorable synthetic accessibility.

### Molecular docking

Virtual screening was conducted using the Maestro program, which incorporated receptor flexibility constraints to enhance accuracy. The program’s sophisticated algorithm effectively predicted the docking poses of the ligands. Three-dimensional coordinates of the target proteins, 4MAN and 1HNJ, were retrieved from the Protein Data Bank (PDB)^41^. The 4MAN^8^ entry, selected for its X-ray resolution of 2.07 Å, and the 1HNJ^42^ entry, chosen for its superior X-ray resolution of 1.46 Å, provided detailed access to their respective active sites.

Each ligand was evaluated with 10 docked poses, and the model with the lowest binding free energy (ΔG) was selected for further analysis. This systematic approach identified the most favorable docking poses, emphasizing key interactions between the ligands and the proteins, thereby facilitating a deeper understanding of their binding mechanisms and potential antitumor activity.

**Fig. 4:**
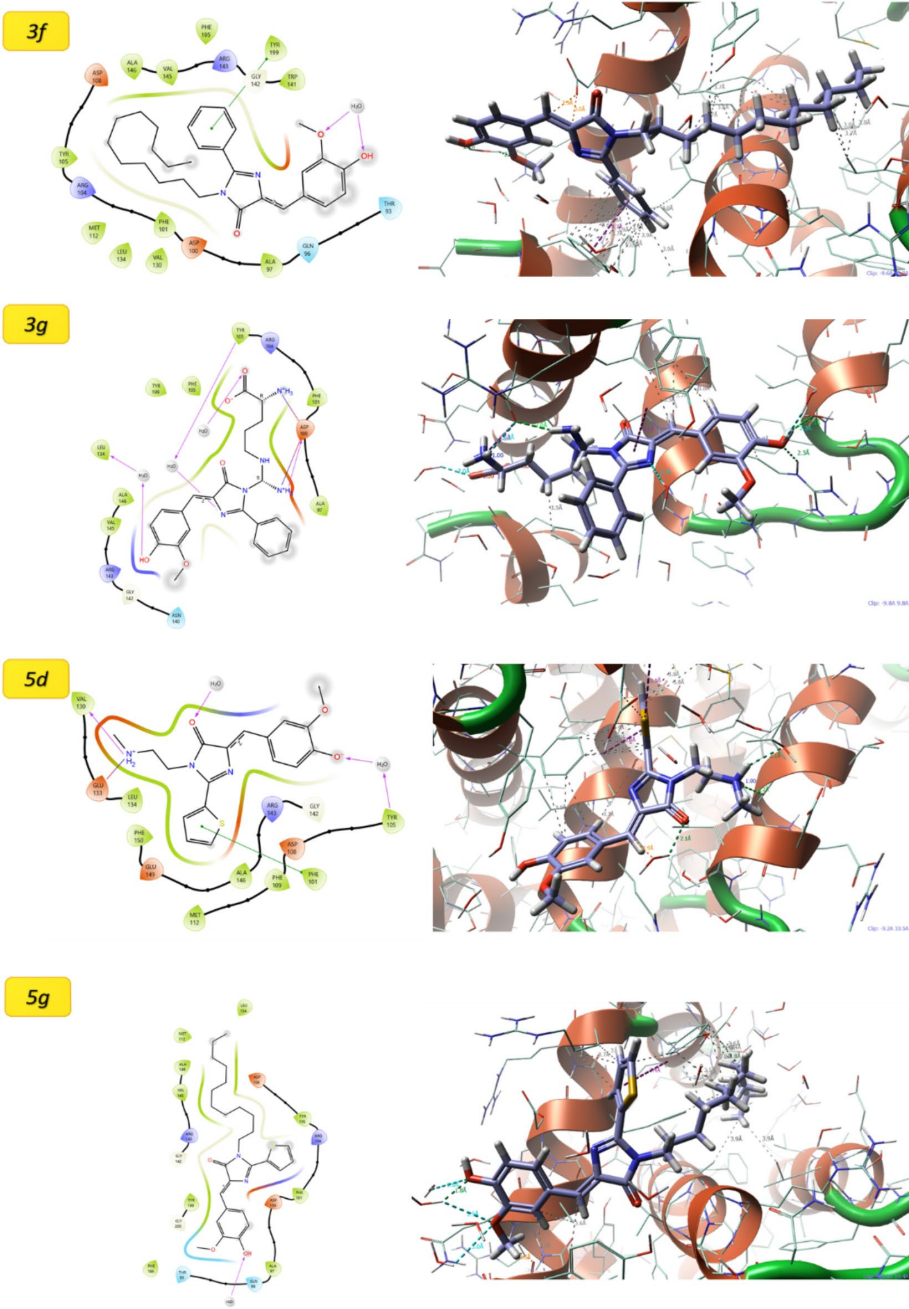
2D and 3D molecular docking poses for the interaction of molecules with 4MAN.

For 4MAN protein (Fig. 4), the molecule 3g exhibits the most favorable interaction, achieving the lowest docking score (− 5.88) as shown in Table 4 and the most negative MMGBSA binding free energy (− 52.13 kcal/mol). The interactions observed for these molecules, as depicted in the 2D interaction poses^34,43–45^, are primarily categorized into three types: Hydrogen Bonding (H-bonding)—involving the formation of directional and specific interactions between hydrogen donors and acceptors (amino acid side chains of proteins), contributing significantly to the stability and specificity of the ligand–protein complex^46,47^. Salt Bridge Interactions—electrostatic interactions between positively and negatively charged groups, playing a crucial role in enhancing binding affinity and ensuring a robust attachment between the ligand and the protein and π–π stacking interactions that interactions occur between aromatic rings in the ligand and the protein, facilitating stable binding through overlap of π-electron clouds. This type of interaction is particularly important for ligands with aromatic moieties, as it enhances the overall binding strength and selectivity. These results strongly suggest high binding affinity and stability of the ligand–protein complex. In contrast, 5d, despite displaying a relatively moderate docking score (− 4.75), demonstrates the least favorable MMGBSA binding energy (− 24.81 kcal/mol), indicative of weaker overall interaction and stability. Molecule 3f achieves a docking score of − 4.40 and a binding free energy of − 44.52 kcal/mol, positioning it as a secondary strong candidate after 3g. On the other hand, 5f shows the weakest docking performance (− 3.71) among the four tested ligands for 4MAN but maintains a moderately favorable MMGBSA binding energy (− 41.89 kcal/mol), indicating a reasonably stable interaction. For 1HNJ protein, molecule 5f demonstrates the strongest interaction, characterized by a docking score of − 4.75 and the most negative MMGBSA binding energy (− 38.63 kcal/mol), highlighting its potential as the top candidate for this target. Molecule 3g, with a docking score of − 4.84 and an MMGBSA binding energy of − 35.88 kcal/mol, shows strong binding affinity and ranks as the second-best candidate. Similarly, molecule 3f achieves a moderate docking score (− 3.37) and a relatively favorable MMGBSA binding energy (− 38.01 kcal/mol), suggesting it as a viable alternative ligand. In contrast, 5d performs the weakest, as evidenced by its highest docking score (− 2.05) and the least negative MMGBSA binding energy (− 14.85 kcal/mol), indicating poor binding affinity and stability. Overall, for 4MAN, molecule 3g emerges as the most promising candidate, while 3f serves as a strong secondary option due to its favorable MMGBSA binding energy. For 1HNJ, molecule 5f is identified as the top-performing ligand, with 3g and 3f being suitable alternatives for further consideration. Across both targets, molecule 5d consistently shows the weakest binding performance, making it less favorable for further investigation.

**Table 4:** Molecular docking results for the studied molecules.

Molecule	Docking score	MMGBSA ΔG Bind
4MAN		
3g	− 5.88	− 52.13
5d	− 4.75	− 24.81
3f	− 4.40	− 44.52
5f	− 3.71	− 41.89
1HNJ		
3g	− 4.84	− 35.88
5d	− 2.05	− 14.85
3f	− 3.37	− 38.01
5f	− 4.75	− 38.63

**Fig. 5:**
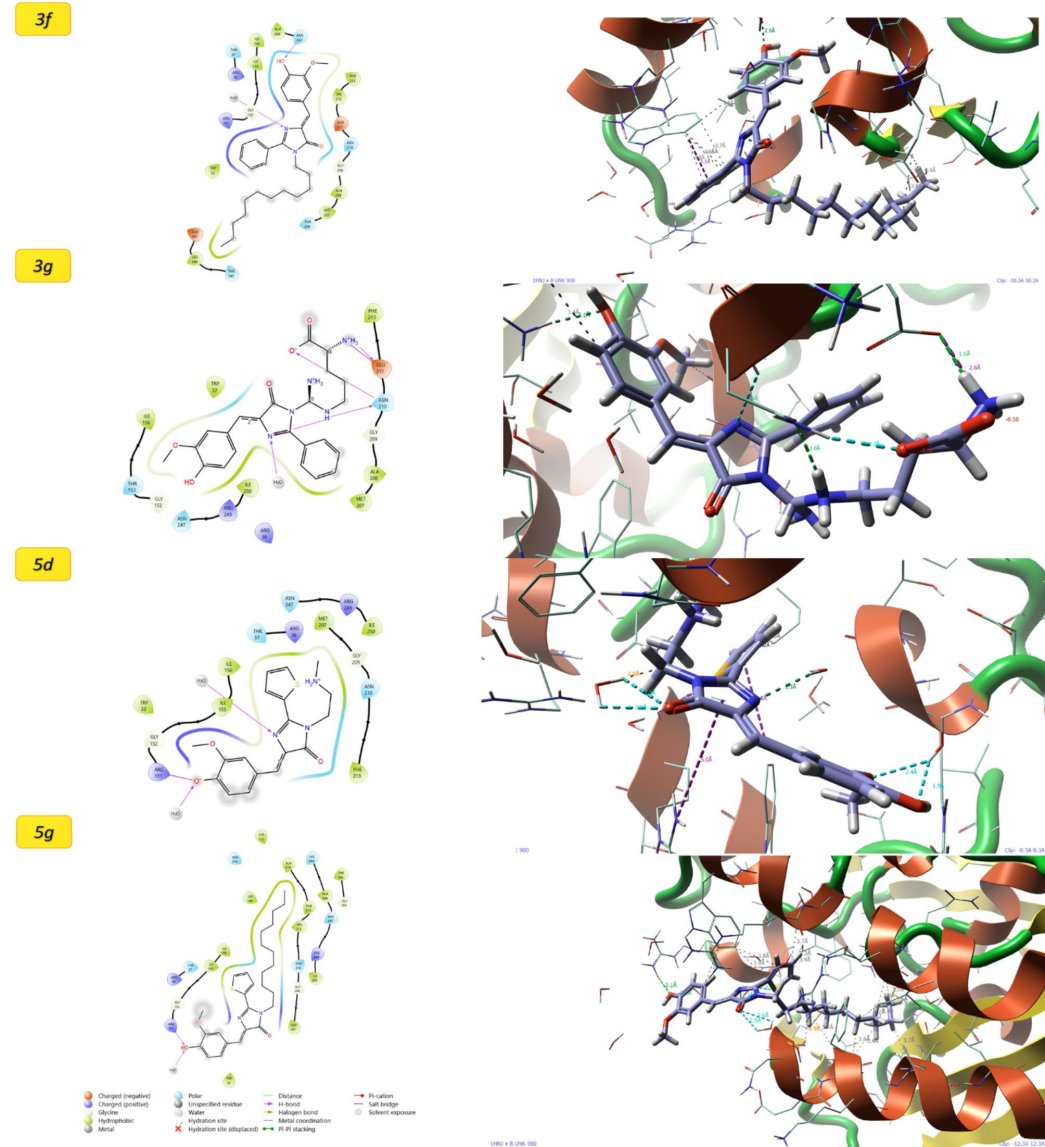
2D and 3D molecular docking poses for the interaction of molecules with 1HNJ.

The interaction data for the protein 1HNJ with various molecules (Fig. 5) reveals significant variations in binding affinity, as indicated by both docking scores and MMGBSA ΔG Bind values. Among the molecules, 3g demonstrates a moderate docking score of − 4.84 and an MMGBSA ΔG Bind value of − 35.88, suggesting a relatively strong binding. Similarly, 5f shows a comparable docking score of − 4.75 but exhibits the strongest MMGBSA ΔG Bind at − 38.63, indicating the most stable interaction. In contrast, 5d has the weakest docking score of − 2.05 and a significantly less favorable binding free energy of − 14.85, implying a weaker interaction with the protein. 3f, while having a moderate docking score of − 3.37, shows a notably strong MMGBSA ΔG Bind of − 38.01, similar to 5f. Overall, 5f and 3f emerge as the top candidates with the strongest binding affinities, as indicated by their more negative MMGBSA ΔG Bind values.

The docking and MMGBSA ΔG Bind data strongly support potential antitumor activity, aligning closely with experimental results, for the molecules interacting with the proteins 4MAN and 1HNJ, which are likely involved in critical cancer-related pathways. Among these, molecules such as 3g, 3f, and 5f stand out due to their consistently strong binding across both proteins, demonstrated by highly favorable docking scores and ΔG Bind values. These interactions suggest their capacity to modulate protein functions or inhibit key pathways essential for tumor progression. Consequently, these molecules emerge as promising lead candidates for therapeutic development, warranting further investigation into their antitumor properties and potential for advancement into targeted cancer therapies.

### Quantitative structure–activity relationship (QSAR) modelling

In this study, we applied Genetic Function Approximation (GFA) to construct predictive models aimed at estimating the IC_50_ values of our synthesized compounds against a panel of cancer cell lines (detailed results are provided in the Supporting Information, GFA1 to GFA4). Ensuring high data quality is fundamental to building robust and meaningful quantitative structure–activity relationship (QSAR) models. Prior to modeling, we carefully validated our dataset, which consisted of the chemical structures of the synthesized compounds, each characterized by 14 well-defined molecular descriptors. The GFA algorithm, which synergistically integrates genetic algorithms with multivariate regression, is particularly effective for uncovering complex, non-linear relationships between molecular features and biological activity^48^. Predicting IC_50_ values with accuracy is a pivotal step in the early stages of drug discovery, especially for identifying and optimizing potential anticancer agents^49^. The resulting GFA models revealed statistically significant correlations between molecular structure and cytotoxic potency, highlighting the value of this approach in prioritizing promising candidates for further preclinical evaluation. The QSAR model, developed using the Genetic Function Approximation (GFA) approach in Materials Studio, predicted the IC_50_ (µM) values of our compounds with remarkable accuracy. The calculations were performed rapidly, demonstrating the efficiency of the model in capturing the underlying structure–activity relationships. This fast and accurate prediction underscores the potential of QSAR modeling as a powerful tool for guiding the early phases of anticancer drug development, enabling rapid screening and prioritization of lead compounds based on their predicted cytotoxic profiles. The following equations were derived from this methodology, yielding precise correlations between the experimental IC_50_ values and those predicted through the structure–activity relationship (SAR) modeling. These models reflect the underlying influence of key molecular descriptors on the compounds biological activities, further validating the robustness and predictive power of the QSAR approach (with R^2^ > 0.95):

**CaCo-2** [Y = 16.729376525 * X2 + 27.800790063 * X13 + 139.637428988 * X21 − 1338.995717909, where: X2 : E : Hydrogen bond acceptor (Fast Descriptors); X13 : U : Subgraph counts (1): path (Fast Descriptors); X21 : (K : Balaban index JY (Fast Descriptors))^2]; **HeLa** [Y = − 1.028007512 * X2 + 193.383429672 * X4 + 5.465175341 * X10 − 2501.509894619, where:X2 : J : Wiener index (Fast Descriptors); X4 : L : Kappa-1 (Fast Descriptors); X10 : (M : Kappa-2 (Fast Descriptors))^2]; **HepG2** [Y = 61.420004311 * X3 + 248.605092774 * X37 + 0.910158629 * X44- 1403.174647551, where: X3 : F : Hydrogen bond acceptor (Fast Descriptors); X37 : (L : Balaban index JX (Fast Descriptors))^2; X44 : (S : Kappa-1 (alpha modified) (Fast Descriptors))^2]; and **MCF-7** [Y = 145.324070428 * X1- 19.976546550 * X19 − 0.000005475 * X24 − 912.550692680where: X1 : D : Rotatable bonds (Fast Descriptors); X19 : (H : AlogP98 (Fast Descriptors))^2; X24 : (M : Wiener index (Fast Descriptors))^2].

## Materials and methods

### Reagents and instruments

Reagents used in this study were purchased from commercial sources and used as received. Glycine, 2-thiophene carbonyl chloride, ethanol amine, butyl amine, dodecyl amine, N-methyl ethylenediamine, 2-chlorophenyl hydrazine hydrochloride, 2-bromophenyl hydrazine hydrochloride, 4-flourophenyl hydrazine hydrochloride, L-arginine, 2-picolylamine and benzoyl chloride.

The melting points were determined using SMP3 apparatus (Stuart Scientific). IR spectra were recorded on Nicolet iS5 FT-IR by Thermo-Fisher Scientific (Waltham, MA, USA). The ^1^H and C-13 NMR were recorded on Bruker Avance, 500 spectrometers (Fällanden, Switzerland) at the University of Jordan (Amman, Jordan) using. Molar masses were determined using MS/MS LCQ Fleet ion trap mass spectrometer (Thermo-Fisher Scientific, Waltham, MA, USA) the analysis was performed in a positive electrospray mode with a voltage of 5.0 kV with a gas flow of 30.0 units, a capillary temperature of 295.0 °C and ionization time of 250.0 ms.

The purification of the prepared compounds was performed by flash chromatography using EtOAc/hexane (6:4) as an eluting solvent.

The ATCC (Manassas, Virginia, USA) provided the HepG2, HeLa, CaCo-2 and MCF-7 cancer cells. The Biological Industries provided Dulbecco’s free Ca^+2^ phosphate-saline buffer (REF # 02-023-1A), Penicillin–Streptomycin solution (catalogue #030311B), and L-glutamine solution (REF # 03-020-1B). Trizma base (Lot SLBF2864V) was provided by Sigma Life Science. MTS Assay was bought from Promega Corporation, Madison, WI, USA. The cell lines were incubated using an Esco CO_2_ cell culture incubator.

### Synthesis and characterization

#### Preparation of benzoyl glycine (1)

A mixture of benzoyl chloride (2.0 g, 14.2 mmol) and glycine (1.0 g, 13.2 mmol) was prepared in a 10 mL water containing NaHCO_3_ (10% by weight) using a 50 mL round bottom flask. The mixture was vigorously stirred until the odor of the benzoyl chloride vanished. Then HCl (10%) was added dropwise to the reaction mixture to neutralize NaHCO_3_. The produced white solid material was collected by filtration under vacuum, rinsed with distilled water, and subjected to recrystallization in boiling water (30 mL). The produced solid was collected by filtration, dried at 65 °C under reduced pressure, and saved for further use. Product weight was 1.7 g (yield 71.4%), m.p 187–189 °C (Lit m.p. 190.0 °C). IR ν cm^−1^: 3340 (secondary amine, N–H stretching), 3068 (O–H stretching, carboxyl group), 2932–2780 (C–H, aliphatic), 1705 (C=O, carboxylic acid), 1665 (C=O, amide) 1602 (C=C, aromatic ring).

^13^C NMR (DMSO) δ in ppm: 172.4 (O=C–OH), 166.8 (N–C=O), 131.4–127.5 (C_6_H_5_–), 41.6 (–C–NH).

#### Preparation of -5(Z)-4-(4-hydroxy-3-methoxybenzylidene)-2-phenyloxazol-5(4H)-one (2)

Vanillin (0.68 g, 4.46 mmol) and benzoyl glycine (prepared above, 0.8 g, 4.46 mmol) were suspended in a round bottom flask (25.0 mL) along with sodium acetate (0.4 g, 5.0 mmol) and acetic anhydride (6.0 mL, 14.0 mmol**)**. The reaction mixture was stirred at 110 °C until vanillin disappeared as detected by TLC (2.5 h, then diluted with 15.0 mL ethanol, and placed in a refrigerator for about 16.0 h. The produced yellow precipitate was collected by suction filtration and dried under reduced pressure at 65 °C. The product mass was 0.90 g (yield 68.70%), m.p 190–192 °C (Lit m.p. 192–193 °C). IR (ν cm^−1^): 3632 (O–H stretching, phenol), 1761 (C=O, lactone), 1655 (C=N, imine), 1606 (C=C, aryl), 1320–1270 (C–O and C–N). ^1^H NMR (DMSO): δ in ppm: 9.80 (1H, s, OH), 8.11 (1H, s, vinylic), 7.59–7.51 (5H, m, aromatic), 7.28–6.94 (3H, m, aromatic), 3.89 (3H, s, methoxy). C-13 NMR (DMSO) δ in ppm: 167.1 (C=O, ester), 163.2 (C=N), 151.1, 142.0, 132.4, (126.2–130.1) (aryl), 56.3 (methyl). *m*/*z*: (M +) for C_17_H_13_NO_4_ Calcd. 295.08, Found 295.13.

#### General protocol for making compounds 3a to g

A solution of amino compound (10 mmol) in tetrahydrofuran (30 mL) containing 2 drops of acetic acid was prepared in a 50 mL round bottom flask. Oxazolone** 2** (10 mmol) was added to the amino acid solution. The produced mixture was refluxed until the complete disappearance of compound **2** as the rection followed by TLC, then placed in a refrigerator until solid stopped precipitating (about 4 h). Produced solid was collected by suction filtration, dried at 60 °C under vacuum, and purified by flash chromatography.

**(Z)-3-((2-bromophenyl)amino)-5-(4-hydroxy-3-methoxybenzylidene)-2-phenyl-3,5-dihydro-4H-imidazol-4-one (3a)**

Imidazolone **3a** was prepared from reacting compound **2** with o-bromoaniline, yield of 69.64%, m.p 98–101 °C and R_f_ 0.12 (Ethyl acetate). IR (ν cm^−1^): 3320 (O–H), 3055 (vinylic), 2930 (C–H, alihatic), 1762 (C=O, amide), 1638 (C=N, imine), 1610 (C=C, aryl), 1320 (C–N), 1224 (C–O), 780 (C–Br). ^1^H NMR (500 MHz, DMSO) δ 10.63 (s, 1H, NH), 9.87 (s, 1, OH), 8.04 (s, 2H), 7.56 (m, 5H), 7.37 (d, 1H), 7.15 (d, 1H), 7.12 (d, 1H), 6.81 (s, 1H), 3.73 (s, 3H, methoxy). ^13^C NMR (DMSO) δ in ppm: 169.2 (C=O, amide), 154.1 (C=N), 148.6 (Ar–OH), 148.0 (Ar–O), 141.7 (=C–N), 138.3 (N–C=O), 135.3–115.1 (C_6_H_5_–), 128.6 (Vinyl), 110.9 (C–Br), 56.2 (O-CH_3_). *m*/*z*: (M +) for C_23_H_17_BrN_2_O_3_ Calcd.: 448.04, Found: 448.12.

**(Z)-3-((2-chlorophenyl)amino)-5-(4-hydroxy-3-methoxybenzylidene)-2-phenyl-3,5-dihydro-4H-imidazol-4-one (3b)**

Imidazolone **3b** was prepared from reacting compound **2** with o-chlororaniline. Brown product, yield of 74.6%, m.p = 79.5–82 °C. R_f_ 0.36 (Hexane/Ethyl acetate, 8:2). IR (ν in cm^−1^): 3320 (O–H), 3053 (vinylic), 2930 (C–H, alipahtic), 1762 (C=O, amide), 1638 (C=N imine,), 1610 (C=C aryl), 1320 (C–N), 1224 (C–O), 760 (C–Cl). ^1^H NMR (500 MHz, DMSO) δ 9.48 (s, 1, OH), 9.27 (s, 1H,), 7.71 (2H, d), 7.60 (s, 1H, vinylic), 7.49–7.39 (m, 5H, aryl), 7.31–7.24 (m, 2H), 7.26 (m, 5H), 7.07 (d, 1H), 6.9 (m, 2H), 3.84 (s, 3H, methoxy). ^13^C NMR (125 MHz, DMSO) δ in ppm:165.5 (C=O, amide), 163.9 (N–C=N), 140.0 (C–OH, phenol), 150.9 (C, aryl), 134.1(Ar-Cl), 132.9–132.0 (=C–N), 132.3–128.4 (C_6_H_5_–), 56.1 (O-CH_3_). *m*/*z*: (M +) for C_23_H_17_ClN_2_O_3_ Calcd.: 404.1, Found: 444.3.

**(Z)-5-(4-hydroxy-3-methoxybenzylidene)-3-(2-hydroxyethyl)-2-phenyl-3,5-dihydro-4H-imidazol-4-one (3c)**

Imidazolone **3c** was prepared from reacting compound **2** with 2-aminoethanol. The beige colored product was obtained in a yield of in a yield of 62.5%, m.p = 167–169 °C. R_f_0.68 (Ethyl acetate). IR (ν in cm^−1^): 3340–3300 (O–H, aliphatic and phenol), 3045 (=C–H), 2932 and 2970 (C–H), 1766 (C=O, lactam), 1634 (C=N), 1610–1580 (C=C), 1320 (C–N), 1224 (C–O).^1^H NMR (500 MHz, DMSO) δ 9.95 (sb, 1H, OH), 7.97 (d, 2H), 7.58 (t, 1H, vinylic), 7.52 (t, 2H), 7.26 (s, 1H), 7.20 (s, 1H), 7.0 (d, 2H), 6.75 (dd, 1H), 3.52 (s, 3H), 3.44 (t, 2H), 3.39 (t, 2H,), 2.38 (s, 1H, OH).^13^C NMR (DMSO) δ in ppm: 169.9 (N–C=O), 156.0 (N–C=N), 148.5 (Ar–OH), 148.7 (Ar–O), 136.5 (=C–N), 134.2–116.7 (C_6_H_5_–), 59.4 (C–OH), 56.2 (O–CH_3_), 45.0 (N–C–).

**(Z)-5-(4-hydroxy-3-methoxybenzylidene)-3-(2-(methylamino)ethyl)-2-phenyl-3,5-dihydro-4H-imidazol-4-one (3d)**

Imidazolone **3d** was prepared from reacting compound **2** with N-methyl ethylenediamine. A pale-yellow product was formed in a 69.3% yield, m.p = 116–118 °C. R_f_ 0.38 (Hexane/Ethyl acetate: 6:4). IR (ν in cm^−1^): 3345–3320 (O–H and N–H, phenol and amine), 3052 (=C–H), 2938 and 2985 (C–H), 1766 (C=O, lactam), 1630 (C=N), 1610–1586 (C=C), 1322 (C–N), 1228 (C–O).^1^H NMR (500 MHz, DMSO) δ 9.4 (s, 1H, OH), 7.75 (m, 3H), 7.49 (s, 1H, vinylic), 7.45 (m, 5H), 7.29 (m, 3H), 6.7 (d, 1H), 3.76 (s, 3H, methoxy), 2.7 (t, 2H), 1.79 (t, 2H), 1.4 (m, 2H)., 1.05 (t, 3H). ^13^C NMR (DMSO) δ in ppm: 169.5 (C=O, amide), 155.7 (C=N imine,), 148.7 (Ar–OH), 148.0 (C, Aryl), 136.6 (=C–N), 134.2–127.3 (Aryl), 56.2 (O–CH_3_), 50.4 (C–N), 42.4 (Ar–C–N), 35.9 (N–CH_3_).

**(Z)-3-butyl-5-(4-hydroxy-3-methoxybenzylidene)-2-phenyl-3,5-dihydro-4H-imidazol-4-one (3e)**

Imidazolone **3e**, was prepared from reacting compound 1 with n-butylamine. A product with a beige color was formed 70.5% yield, m.p = 177.5–180 °C. R_f_ 0.10 (Hexane/Ethyl acetate: 6:4). IR (ν in cm^−1^): 3335 (O–H phenol), 3050 (vinylic), 2934 and 2975 (C–H, aliphatic), 1762 (C=O, amide), 1634 (C=N), 1612–1590 (C=C), 1320 (C–N), 1222 (C–O).^1^H NMR (500 MHz, DMSO) δ 10.09 (s, 1H, OH), 8.21 (s, 1H), 8.06 (d, 2H), 7.57 (s, 1H), 7.52 (d, 2H), 7.36 (s, 1H), 7.27 (s, 1H), 7.18 (s,1H), 7.16 (s, 1H), 7.06 (t, 1H), 3.56 (s, 3H, methoxy), 3.17 (t, 2H), 1.46 (m, 2H), 1.31 (m, 2H)., 0.88 (t, 3H). ^13^C NMR (DMSO) δ in ppm:169.8 (N–C=O), 156.0 (C=N), 148.5 (Ar–OH), 148.8 (C–O, aryl), 136.4 (=C–N), 134.0–126.3 (Aryl), 56.1 (O–CH_3_), 41.8 (–C–N), 30.4 (C–), 20.3 (–C-Methyl), 13.7 (CH_3_).

**(Z)-3-dodecyl-5-(4-hydroxy-3-methoxybenzylidene)-2-phenyl-3,5-dihydro-4H-imidazol-4-one (3f)**

Imidazolone **3f** was prepared from reacting compound **2** with n-dodecyl amine. The orange product was formed in a 64.13% yield, m.p = 109.5–111 °C. R_f_ 0.23 (Hexane/Ethyl acetate: 5:5). IR (ν in cm^−1^): 3330 (O–H phenol), 3035 (vinylic), 2930 and 2982 strong (C–H, aliphatic), 1766 (C=O, amide), 1631 (C=N), 1610–1592 (C=C, aryl), 1326 (C–N), 1220 (C–O). ^1^H NMR (500 MHz, DMSO) δ 9.95 (s, 1H, OH), 8.08 (s, 1H), 7.97 (s, 1H, vinylic), 7.76 (s, 1H), 7.50 (d, 2H), 7.21 (s, 12H), 6.97 (s, 1H), 6.73 (t, 2H), 3.80 (s, 3H, methoxy), 3.48(t, 2H), 1.24–1.77 (m, 20H), 0.86 (t, 3H). ^13^C NMR (DMSO) δ in ppm:175.8 (C=O), 168.6 (C=O, amide), 155.9 (C=N), 148.6 (Ar–OH), 147.8 (Ar–O), 135.5 (=C–N), 134.0–116.3 (C_6_H_5_–), 56.2 (O–CH_3_), 41.5 (–C–N), 31.8–26.7 (–C–), 22.3 (–C-Methyl), 14.0 (CH_3_).

**(Z)-2-amino-5-((amino(4-(4-hydroxy-3-methoxybenzylidene)-5-oxo-2-phenyl-4,5-dihydro-1H-imidazol-1-yl)methyl)amino)pentanoic acid (3g)**

The amino acid L-arginine (3.0 mmol) was suspended in methanol (30 mL), followed by the addition of oxazolone 2 (3.0 mmol). The mixture was stirred vigorously at room temperature for 24 h. Then, the solvent was removed under vacuum, and the product was washed with water, then methanol, and diethyl ether. A beige colored product obtained in a 61.76% yield, m.p 160–162 °C. R_f_ 0.21(Ethyl acetate). ^13^C NMR (125 MHz, DMSO) δ in ppm:171.0 (N–C=O), 158.4 (C=N), 148.9 (Ar–OH), 148.7 (Ar–O), 138.4 (=C–N), 134.1–125.3 (C_6_H_5_–), 82.9 (N–C–N), 55.95 (O–CH_3_), 55.1 (–C–NH_2_), 44.1 (–C–NH), 25.8 (–C–).

#### Preparation of 2-thiophenecarbonylglycine

A mixture of 2-thiophenecarbonyl chloride (2.0 g, 14.0 mmol) and glycine (1.1 g, 14.0 mmol) was prepared in a 10% solution of sodium bicarbonate (10.0 mL). The mixture was hand shaken until the odor of the 2-thiophenecarbonyl chloride disappeared. Then it was neutralized by the dropwise addition of HCl (10%), the produced solid material was collected by filtration under vacuum, rinsed with distilled water, and recrystallized in boiling water (30 mL). The white product of 2-thiophenecarbonylglycine was collected by suction filtration and dried at 65 °C in an oven under reduced pressure. The mass of the product was 1.7 g (yield 69.67%), m.p 121–123 °C. IR ν cm^−1^: 3335 (N–H, stretching), 3268 (O–H carboxyl group, stretching), 2932–2890 (C–H, aliphatic), 1704 (C=O, carboxylic acid), 1662 (C=O, amide) 1595 (C=C, thiophene ring). ^13^C NMR (DMSO) δ in ppm: 172.8 (C=O, carboxyl), 164.3 (amide), 141.2 (=C–S),131.2–127.8 (Ar), 41.4 (–C–N).

#### Preparation of (Z)-4-(4-Hydroxy-3-Methoxybenzylidene)-2- (thiophen-2-yl)oxazol-5-(4H)-One (4)

A sample of vanillin (0.76 g, 5.0 mmol) was suspended in a 50 mL round bottom flask containing acetic anhydride (6.1 mL, 15.0 mmol**)** and sodium acetate anhydrous (0.8 g, 10.0 mmol). 2-thiophenecarbonylglycine (0.93 g, 5.0 mmol) was added to the mixture. The reaction mixture was stirred at 110 °C until vanillin was consumed, as shown by TLC (3.0 h). The reaction mixture was diluted with 15.0 mL ethanol and placed in a refrigerator for about 16.0 h. The produced yellow precipitate was filtered, rinsed two times with ethyl ether, and dried at 65 °C under vacuum. The product mass was 1.02 g (yield 67.70%), m.p 199–201 °C. IR (ν cm^−1^): 3350 (O–H stretching, phenol), 1768 (C=O, lactone), 1652 (C=N), 1603 (C=C), 1318–1268 (C–O and C–N). ^1^H NMR (DMSO): δ in ppm: ^1^H NMR (500 MHz, DMSO) δ 9.3 (s, 1H, OH), 8.11 (t, 2H), 7. 94(d, 1H), 7.80 (d, 1H), 7.53 (t, 1H), 7.26 (s, 1H), 7.24 (d, 1H), 3.74, (s, 3H)). C-13 NMR (DMSO) δ in ppm: 167.1 (C=O, ester), 162.2 (C=N), 151.1 (Ar–O), 142.0 (Ar–OH), 134.4 (=C–N), (132.6–126.2) (aryl), 56.2 (O-CH_3_). *m*/*z*: (M +) for C_15_H_11_NO_4_S Calcd. 301.3, Found 301.1, M + 2 303.1.

#### General protocol for making compounds 5a to i

In a round bottom flask (25.0 mL), a solution of compound **4** and an amine (10 mmol) in THF (25 mL) prepared, Four drops of CH_3_COOH were added to the solution and refluxed until the complete disappearance of compound **4** as shown by TLC. The reaction mixture was mixed with ice (25g) and placed in the refrigerator until solid stopped forming (2 h), then it was filtered and solid was collected, dried at 60 °C under vacuum, and purified by flash chromatography.

**(Z)-3-((2-bromophenyl)amino)-5-(4-hydroxy-3-methoxybenzylidene)-2-(thiophen-2-yl)-3,5-dihydro-4H-imidazol-4-one (5a)**

Imidazolone 5a was prepared from reacting compound 4 with o-bromoaniline, the brown product was formed in 63.40% yield, m.p 160–162 °C. R_f_ 0.23 (Hex/EtOAc 6:4). IR (ν cm^−1^): 3322 (O–H), 3057 (=C–H), 1763 (C=O, amide), 1641 (C=N, imine), 1604 (C=C, aryl), 1322 (C–N), 1227 (C–O), 782 (C–Br). ^1^H NMR (500 MHz, DMSO) δ 9.48 (s, 1, OH), 9.27 (s, 1H, N–H), 7.71 (2H, d, J = 8.6 Hz), 7.6 (s, 1H, vinylic), 7.39–7.48 (m, 5H), 7.20–7.30 (m, 2H), 7.26 (m, 5H), 7.10 (d, 1H), 6.9 (m, 2H), 3.87 (s, 3H, OMe). ^13^C NMR (DMSO) δ in ppm: 167.7 (C=O, amide), 148.7 (Ar-OH), 148.5 (Ar–O), 146.7 (C=N), 142.4 (=C–NH), 138.5 (=C–S), 138.2 (C–N), 133.4–115.7 (Ar), 111.3 (C–Br), 56.3 (O-CH_3_). *m*/*z*: (M +) for C_21_H_16_BrN_2_O_3_S Calcd.: 469.01, Found: 462.21, (M + 1 470.32).

**(Z)-3-((2-chlorophenyl)amino)-5-(4-hydroxy-3-methoxybenzylidene)-2-(thiophen-2-yl)-3,5-dihydro-4H-imidazol-4-one (5b)**

Imidazolone 5b was prepared from reacting compound 4 with and o-chloroaniline produces a brown colored compound 5b in a yield of 71.36%, m.p 92–94 °C. R_f_ 0.52 (Hex/EtOAc 4:2). IR (νcm^−1^): 3326 (O–H, phenol), 3057 (vinylic), 1761 (C=O), 1640 (C=N), 1603 (C=C), 1322 (C–N), 1218 (C–O). ^1^H NMR (500 MHz, DMSO) δ 9.48 (s, 1, OH), 9.27 (s, 1H, N–H), 7.71 (2H, d), 7.6 (s, 1H, vinylic), 7.40–7.50 (m, 5H), 7.20–7.30 (m, 2H), 7.26 (m, 5H), 7.07 (d, 1H), 6.9 (m, 2H), 3.87 (s, 3H, methoxy). ^13^C NMR (DMSO) δ in ppm:167.8 (C=O, amide), 148.9 (Ar-OH), 148.1 (Ar–O), 146.7 (N–C=N), 138.5 (=C–S), 138.7 (=C–NH), 130.3–125.4 (Ar), 122.0 (C–Cl), 131.27–128.37 (C_6_H_5_-), 56.2 (O-CH_3_). *m*/*z*: (M +) for C_21_H_16_ClN_2_O_3_S Calcd.: 425.01, Found: 425.11, (M + 2 427.32).

**(Z)-5-(4-hydroxy-3-methoxybenzylidene)-3-(2-hydroxyethyl)-2-(thiophen-2-yl)-3,5-dihydro-4H-imidazol-4-one (5c)**

Imidazolone 5c was prepared from reacting compound 4 with aminoethanol, the beige product was acquired in a 67.62% yield, m.p 117.5–119 °C. R_f_ 0.33 (EtOAc neat). IR (ν cm^−1^): 3330 (O–H), 3051 (vinylic), 2932 (C–H, aliphatic), 1758 (C=O, amide), 1633 (C=N, imine), 1605 (C=C, aryl), 1322 (C–N), 1221 (C–O). ^1^H NMR (500 MHz, DMSO) δ 9.83 (s, 1, OH), 8,22 (s, 1H), 7.97 (s, 1H, vinylic), 7.83 (s, 1H), 7.21 (s, 2H)), 7.00 (d, 1H), 6.75 (d, 1H), 3.76 (t, 2H), 3.72 (s, 3H, methoxy), 3.23 (t, 2H), 3.12 (s, 1H, OH). ^13^CNMR (DMSO) δ in ppm: 168.0 (N–C=O), amide, 161.8 (C=N, imine), 148.5 (Ar–OH), 148.7 (Ar–O), 136.9 (=C–S),136.4 (=C–N), 130.2–115.7 (Ar), 59.4 (C–OH), 56.2 (O-CH_3_), 45.0 (N–C–). *m*/*z*: (M +) for C_17_H_16_N_2_O_4_S Calcd.: 344.10, Found: 344.15.

**(Z)-5-(4-hydroxy-3-methoxybenzylidene)-3-(2-(methylamino)ethyl)-2-(thiophen-2-yl)-3,5-dihydro-4H-imidazol-4-one (5d)**

Imidazolone 5d was prepared from reacting compound 4 with N-methyl ethylenediamine, a brown solid was achieved in a 64.7% yield, m.p 79–81 °C. R_f_ 0.54 (EtOAc neat). IR (νcm^−1^): 3340–3330 (N–H and O–H), 3048 (vinylic), 2941 (C–H, aliphatic), 1756 (C=O, amide), 1631 (C=N, imine), 1604 (C=C), 1320 (C–N), 1220 (C–O). ^1^H NMR (500 MHz, DMSO) δ 9.32 (s, 1H, OH), 7.6 (d, 1H), 7.52 (s, 1H, vinylic), 7.43 (dd, 1H), 7.29 (m, 3H), 7.15 (t, 1H), 6.87 (d, 1H), 4.08 (t, 2H), 3.82 (s, 3H, methoxy), 2.91 (t, 2H), 2.45 (d, H), 1.66, (t, 1H, NH). ^13^CNMR (DMSO) δ in ppm: 167.7 (C=O, amide), 155.9 (C=N, imine), 148.7 (Ar–OH), 148.0 (Ar–O), 136.9 (=C–N), 136.4 (=C–S), 130.9–115.1 (Ar), 56.2 (O–CH_3_), 50.4 (–C–N), 42.4 (Ar–C–N), 35.9 (N-CH_3_). *m*/*z*: (M +) for C_18_H_19_N_3_O_3_S Calcd.: 357.43, Found: 357.15.

**(Z)-3-butyl-5-(4-hydroxy-3-methoxybenzylidene)-2-(thiophen-2-yl)-3,5-dihydro-4H-imidazol-4-one (5e)**

Imidazolone 5e was prepared from reacting compound 4 with n-butyl amine, the white product was obtained in 68.17% yield, m.p 171–173 °C. R_f_ 0.16 (Hex/EtOAc: 6:4). IR (ν cm^−1^): 3334 (O–H), 3048 (=C–H, vinylic), 2942 (C–H, aryl), 1752 (C=O, amide), 1633 (C=N, imine), 1602 (C=C, aryl), 1321 (C–N), 1222 (C–O). ^1^H NMR (500 MHz, DMSO) δ 9.74 (s, 1, OH), 8.51 (s, 1H), 8.08 (s, 1H, vinylic), 7.82 (s, 1H), 7.40 (s, 1H), 7.27 (m, 2H), 7.16 (d, 1H), 7.08 (d, 1H), 3.57 (s, 3H, OMe), 3.16 (t, 3H), 1.46 (t, 2H), 1.32 (m, 2H), 0.91 (t, 3H). ^13^CNMR (DMSO) δ in ppm:167.8 (C=O, amide), 156.1 (C=N), 148.5 (Ar-OH), 148.8 (Ar–O), 136.9 (=C–N), 136.3 (= C-S), 130.0–115.3 (Ar), 56.2 (O–CH_3_), 41.8 (–C–N), 30.5 (–C–), 20.2 (–C-Methyl), 13.8 (CH_3_). *m*/*z*: (M +) for C_19_H_20_N_2_O_3_S Calcd.: 355.44, Found: 356.23.

**(Z)-3-dodecyl-5-(4-hydroxy-3-methoxybenzylidene)-2-(thiophen-2-yl)-3,5-dihydro-4H-imidazol-4-one (5f)**

Imidazolone 5f was prepared from reacting compound 4 with n-dodecyl amine, the pale-yellow product was obtained in 72.55% yield, m.p 133–134.5 °C. R_f_ 0.21 (Hex/EtOAc: 6:4). IR (νcm^−1^): 3330 (O–H, aryl), 3042 (vinylic), 2936 and 2868 (C–H, aliphatic), 1750 (C=O, amide), 1631 (C=N, imine), 1601 (C=C, aryl), 1323 (C–N), 1222 (C–O). ^1^H NMR (500 MHz, DMSO) δ 9.34 (s, 1, OH), 7.57 (d, 1H), 7.53 (s, 1H, vinylic), 7.38 (dd, 1H), 7.31 (m, 3H), 7.13 (t, 1H), 6.83 (d, 1H), 3.83 (s, 3H, OMe), 3.72 (t, 3H), 1.73 (t, 2H), 1.41 (m, 2H), 0.93 (t, 3H). ^13^CNMR (DMSO) δ in ppm:167.8 (O=C–N), 156.6 (N–C=N), 148.6 (Ar–OH), 147.8 (Ar–O), 136.9 (=C–N), 136.3 (=C–S), 130.9–115.3 (Ar), 56.2 (O–CH_3_), 41.9 (–C–N), 31.8–26.5 (–C–), 22.7 (–C-Methyl), 14.1 (CH_3_). *m*/*z*: (M +) for C_27_H_36_N_2_O_3_S Calcd.: 368.66.44, Found: 368.78.

**(Z)-5-(4-hydroxy-3-methoxybenzylidene)-3-(pyridin-3-ylmethyl)-2-(thiophen-2-yl)-3,5-dihydro-4H-imidazol-4-one (5g)**

Imidazolone 5g was prepared from reacting compound 4 with picolamine, the beige product was formed in a 73.06% yield, m.p 101–103 °C. R_f_ 0.71 (Ethyl acetate neat). IR (νcm^−1^): 3332 (O–H), 3042 (vinylic), 2936 and 2868 (C–H, aliphatic), 1750 (C=O, amide), 1631 (C=N imine), 1601 (C=C, aryl), 1323 (C–N), 1222 (C–O). ^1^H NMR (500 MHz, DMSO) δ 9.36 (s, 1, OH), 8.50 (bs, 1H), 7.89 (bs, 1H), 7.54 (d, 1H), 7.44 (s, 1H, vinylic), 7.41 (dd, 1H), 7.25 (m, 3H), 6.85 (d, 1H), 5.27 (s, 2H), 3.85 (s, 3H, methoxy). ^13^CNMR (DMSO) δ in ppm: 168.6 (C=O, amide), 152.0 (C=N), 149.5 (C=N), 148.6 (Ar-OH), 147.8 (Ar–O), 136.9 (=C–N), 136.3 (=C–S), 130.9–115.3 (Ar), 56.2 (O–CH_3_), 41.9 (–C–N), 31.8–26.5 (–C–), 22.7 (–C-Methyl), 14.1 (CH_3_). *m*/*z*: (M +) for C_27_H_36_N_2_O_3_S Calcd.: 391.45, Found: 391.76.

### Anticancer study

#### Cell lines

The synthesized derivatives were tested against HepG2, HeLa, CaCo-2, and MCF-7.

#### Cytotoxicity test

75 cm^2^ polycarbonate plates filled with culture growth medium (CGM) were used to cultivate the cancer cells. The CGM was made up of DMEM medium augmented with 10% (FBS), pen/strep, and L-glutamine. To promote cell proliferation, the plates were kept at 37 °C in a humidified atmosphere with 5% CO_2_.

Following confluency, cells underwent two cycles of washing with 15 mL of Calcium free phosphate-buffered saline (PBS). The cells were then treated with 1 mL of trypsin, and the plate was incubated for about 3 min. Then, ten milliliters of CGM were added to the plate to make the trypsin inactive. After that, the cell suspension was gathered and made diluted. A 96-well plate was filled with the diluted cell solution, and it was allowed to adhere for twenty-four hours. Following that, 100 μL of the compounds dissolved in 1% DMSO, in a concentration range from 62.5 to 500 µM, together with a control and blank, were added and the plate was left incubated for 48 h.

Finally, in each well a 20 μL of MTS solution was added and left in the incubator for 2 h. A plate reader was used to determine the absorbance of each well.

#### Statistical analysis

The anticancer activity was performed in triplicate and demonstrated as the means ± standard deviation. All graphs and the measurement of the IC_50_ were carried out by Graph-Pad Prism Software 9.

## Conclusions

Several new imidazolone derivatives with various substituents were synthesized using the natural product vanillin as starting material. In terms of anticancer activity, imidazolones with lipophilic chains and pyridyl groups showed to be promising molecular targets for developing anticancer drugs. They displayed remarkable activities against various cancer cells. The ADME study validated drug-likeness and moderate bioavailability for all examined molecules, with molecule 5d exhibiting the most straightforward synthesis route. Molecular docking demonstrated robust binding interactions, identifying molecules 3g and 5f as the most promising options for targeted cancer therapy owing to their elevated binding affinities and stability with both 4MAN and 1HNJ proteins. A QSAR study revealed statistically significant correlations between molecular structure and cytotoxic potency. These results show the potential of the compounds as prospective anticancer drugs. Additional biochemical and pharmacological research is being conducted to enhance their pharmacological profile and investigate the precise mechanism of action.

## Electronic supplementary material

Below is the link to the electronic supplementary material.

Supplementary Material 1

Supplementary Material 2

## Data Availability

All data generated or analysed during this study are included in this published article and its supplementary information files.
