# Supplementary material for: Design, synthesis, anticancer properties, and molecular docking of imidazolone derivatives with lipophilic moiety
Source: Sci Rep. 2025 May 27;15:18537. doi: 10.1038/s41598-025-97478-2 (PMC12117022; doi:10.1038/s41598-025-97478-2)
Supplement: Supplementary file 1 — Supplementary Material 1 [file 41598_2025_97478_MOESM1_ESM.docx]

**Design, Synthesis, Anticancer Properties, and Molecular Docking of Imidazolone Derivatives with Lipophilic Moiety**

**Oswa Fares^1^, Othman Hamed^1^*, Mohyeddin Assali^2^*, Avni Berisha^3,4*^, Haythem Saadeh^5^, Bahia Abu Lail^5^, Omar Dagdag^6^, Abdullah Samaro**^7^**, Waseem Mansour^1^, Nidal Jaradat^2^, Saber Abu-Jabal^1^**

^1^ Department of Chemistry, Faculty of Science, An-Najah National University, P.O. Box 7, Nablus, Palestine

^2^ Department of Pharmacy, Faculty of Medicine and Health Sciences, An-Najah National University, P.O. Box 7, Nablus, Palestine

^3^ Department of Chemistry, Faculty of Natural and Mathematics Science, University of Prishtina, 10000 Prishtina, Kosovo; avni.berisha@uni-pr.edu

^4^ Materials Science-Nanochemistry Research Group, Nano Alb-Unit of Albanian Nanoscience and Nanotechnology, 1000 Tirana, Albania

^5^ Department of Chemistry, The University of Jordan, Amman, Jordan

^6^ Department of Mechanical Engineering, Gachon University, Seongnam 13120, Republic of Korea

^7^ Department of Biomedical Sciences, Faculty of Medicine and Health Sciences, An-Najah National University, P.O. Box 7, Nablus, Palestine

* Correspondence: ohamed@najah.edu; m.d.asssali@najah.edu; [avni.berisha@uni-pr.edu](mailto:avni.berisha@uni-pr.edu)

**Fig.S1:**Imidazolones 3a to g viabilities against the tested cancer cells.

**Fig.S2:** Imidazolones 5a to 5g viabilities against the tested cancer cells


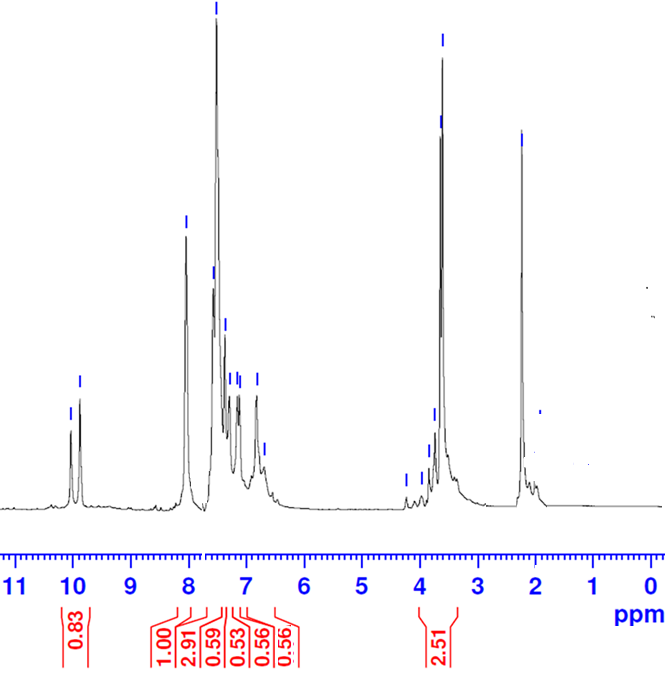


**Fig. S3a:** ^1^H NMR of (Z)-3-((2-bromophenyl)amino)-5-(4-hydroxy-3-methoxybenzylidene)-2-phenyl-3,5-dihydro-4H-imidazol-4-one **(3a).**


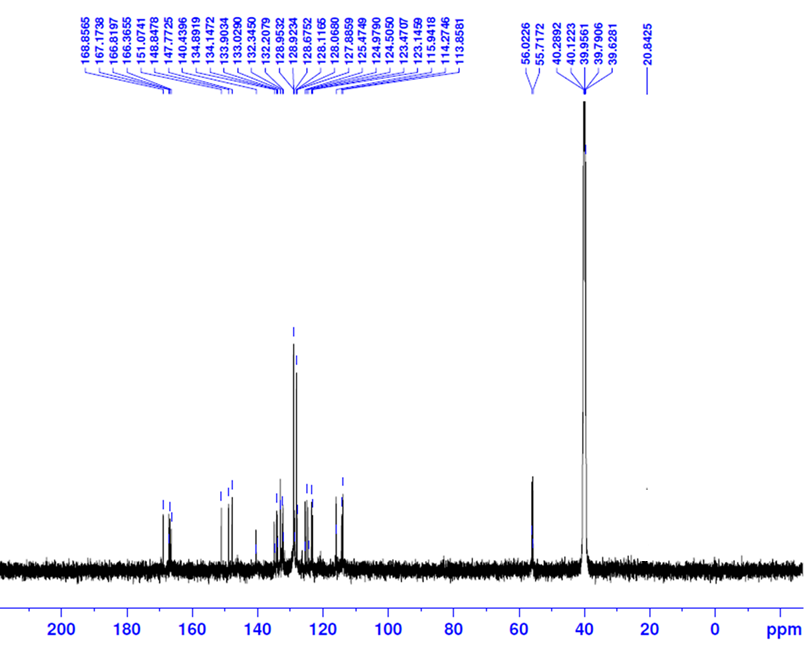


**Fig. S3b** ^13^C NMR of (Z)-3-((2-bromophenyl)amino)-5-(4-hydroxy-3-methoxybenzylidene)-2-phenyl-3,5-dihydro-4H-imidazol-4-one **(3a).**


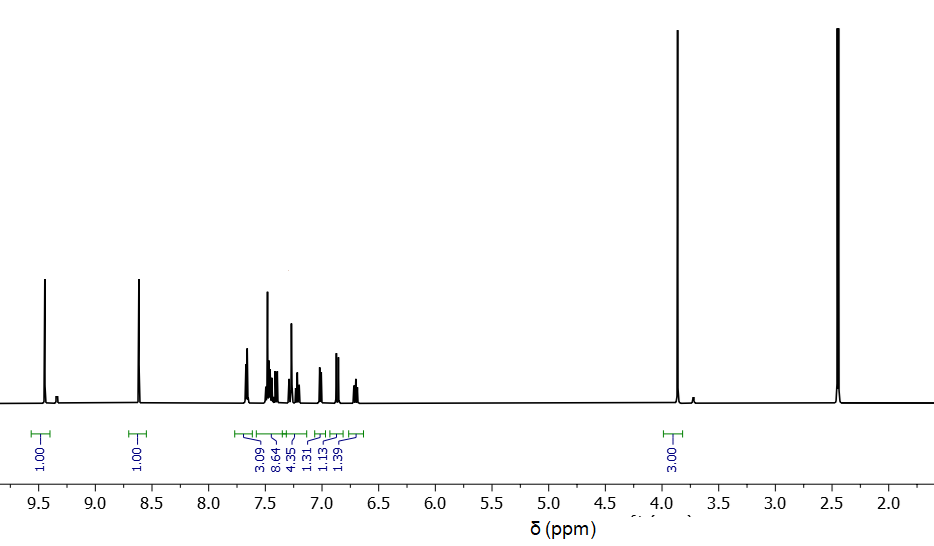


**Fig. S4:** ^1^H NMR of (Z)-3-((2-chlorophenyl)amino)-5-(4-hydroxy-3-methoxybenzylidene)-2-phenyl-3,5-dihydro-4H-imidazol-4-one **(3b).**


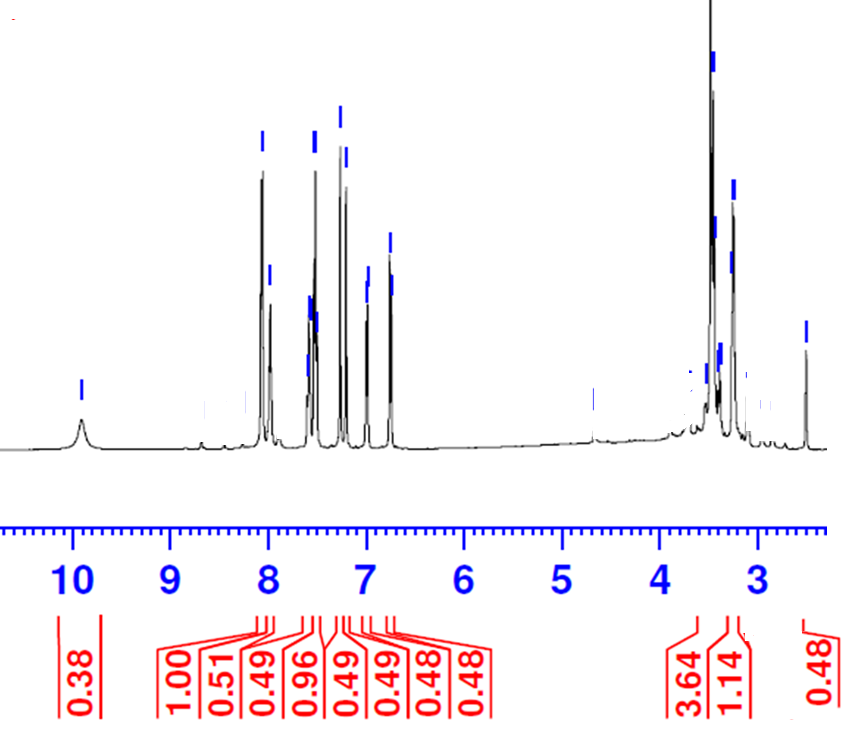


**Fig. S5a:** ^1^H NMR of (Z)-5-(4-hydroxy-3-methoxybenzylidene)-3-(2-hydroxyethyl)-2-phenyl-3,5-dihydro-4H-imidazol-4-one **(3c)**.


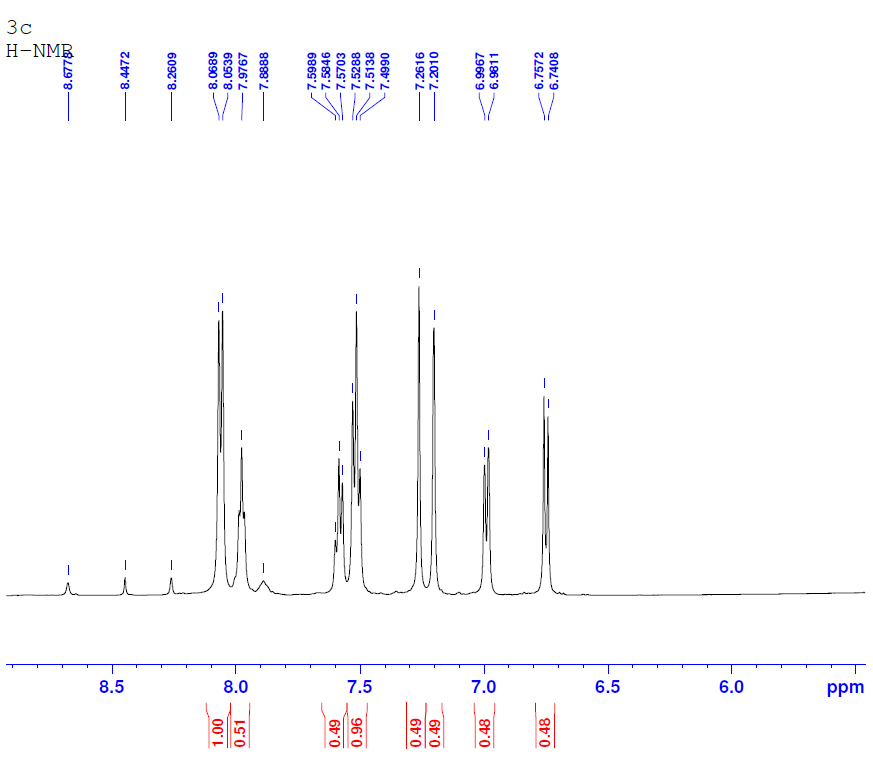


**Fig. S5b:** ^1^H NMR of (Z)-5-(4-hydroxy-3-methoxybenzylidene)-3-(2-hydroxyethyl)-2-phenyl-3,5-dihydro-4H-imidazol-4-one **(3c)**.


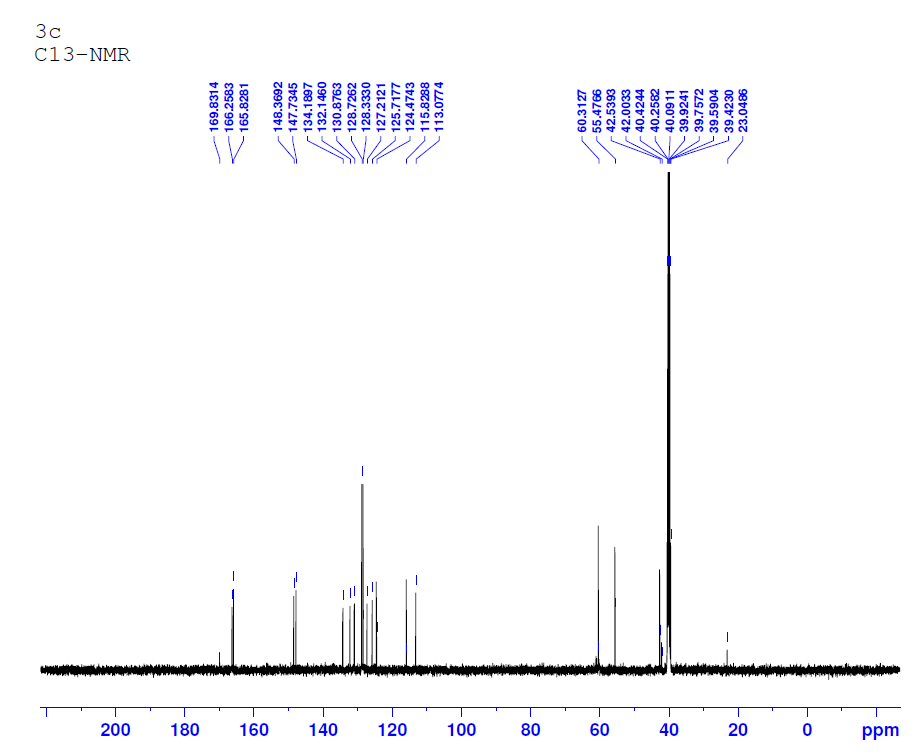


**Fig. S5c:** ^13^C NMR of (Z)-5-(4-hydroxy-3-methoxybenzylidene)-3-(2-hydroxyethyl)-2-phenyl-3,5-dihydro-4H-imidazol-4-one **(3c)**.


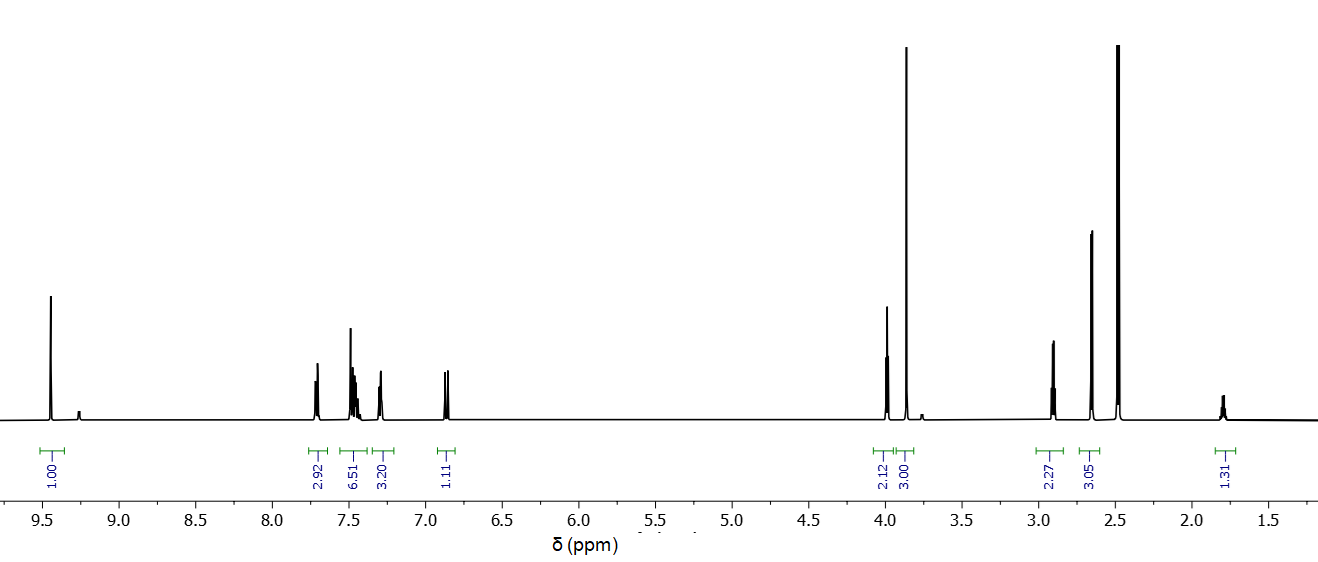


**Fig. S6a:** ^1^H NMR of (Z)-5-(4-hydroxy-3-methoxybenzylidene)-3-(2-(methylamino)ethyl)-2-phenyl-3,5-dihydro-4H-imidazol-4-one **(3d)**.


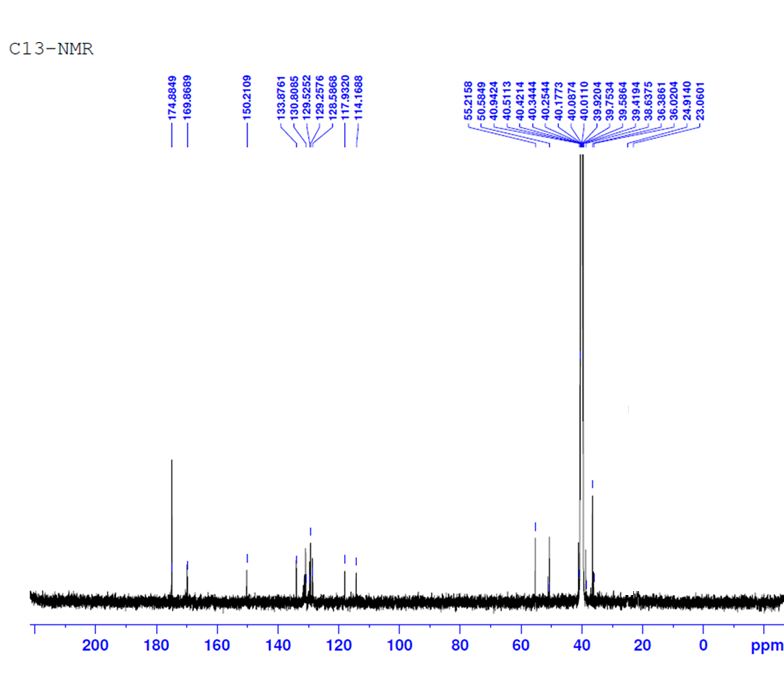


**Fig. S6b:** ^13^C NMR of (Z)-5-(4-hydroxy-3-methoxybenzylidene)-3-(2-(methylamino)ethyl)-2-phenyl-3,5-dihydro-4H-imidazol-4-one **(3d)**.


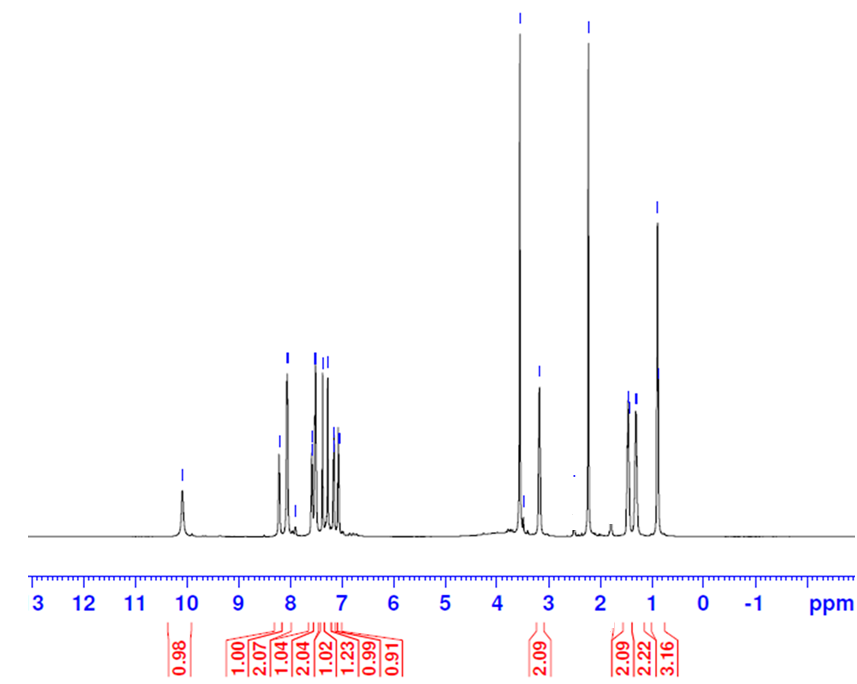


**Fig. S7a:** ^1^H NMR of (Z)-3-butyl-5-(4-hydroxy-3-methoxybenzylidene)-2-phenyl-3,5-dihydro-4H-imidazol-4-one **(3e)**.


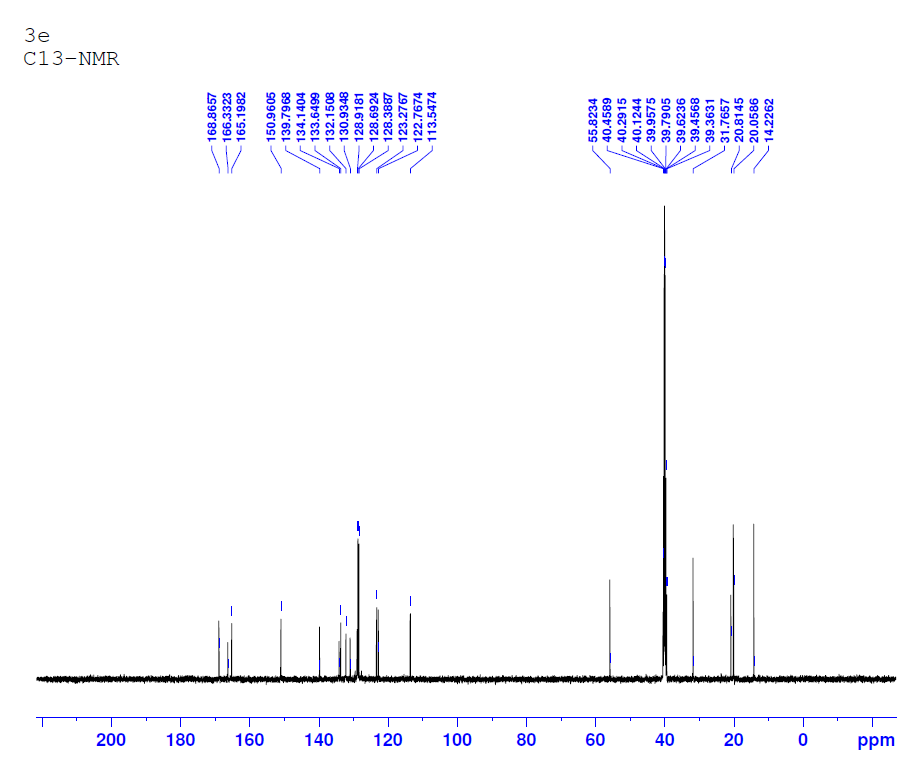


**Fig. S7b:** ^13^C NMR of (Z)-3-butyl-5-(4-hydroxy-3-methoxybenzylidene)-2-phenyl-3,5-dihydro-4H-imidazol-4-one **(3e)**.


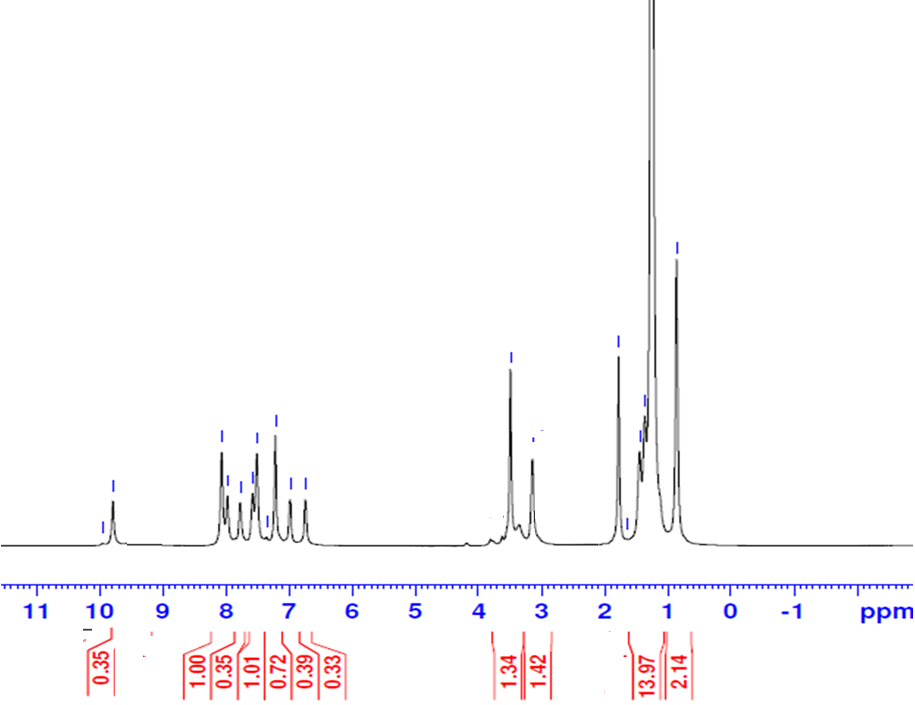


**Fig. S8a:** ^1^H NMR of (Z)-3-dodecyl-5-(4-hydroxy-3-methoxybenzylidene)-2-phenyl-3,5-dihydro-4H-imidazol-4-one **(3f).**

**
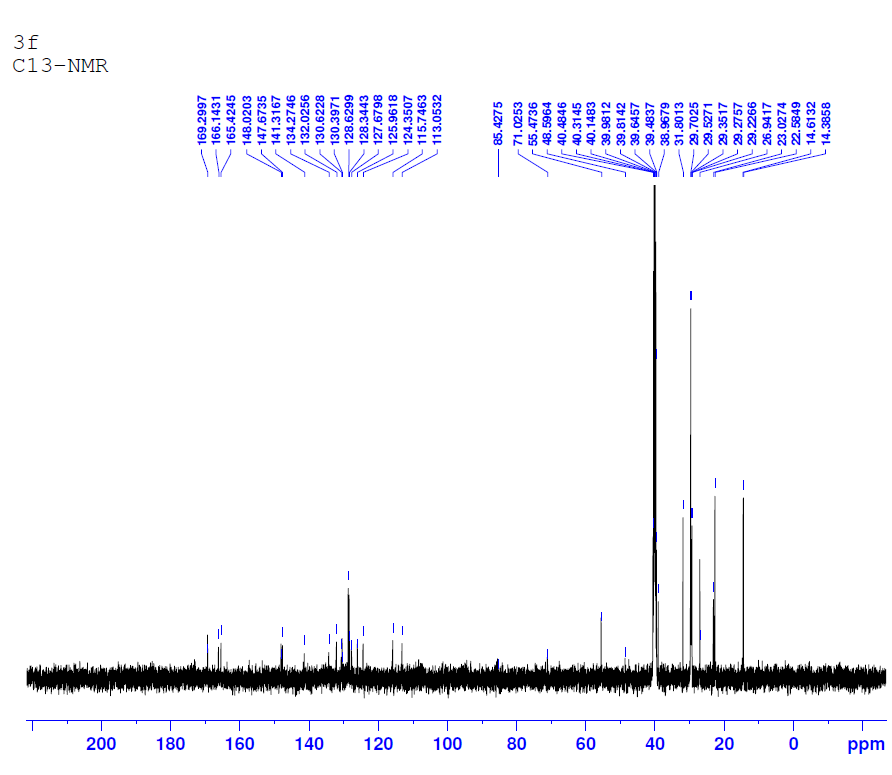
**

**Fig. S8b:** ^13^C NMR of (Z)-3-dodecyl-5-(4-hydroxy-3-methoxybenzylidene)-2-phenyl-3,5-dihydro-4H-imidazol-4-one **(3f).**


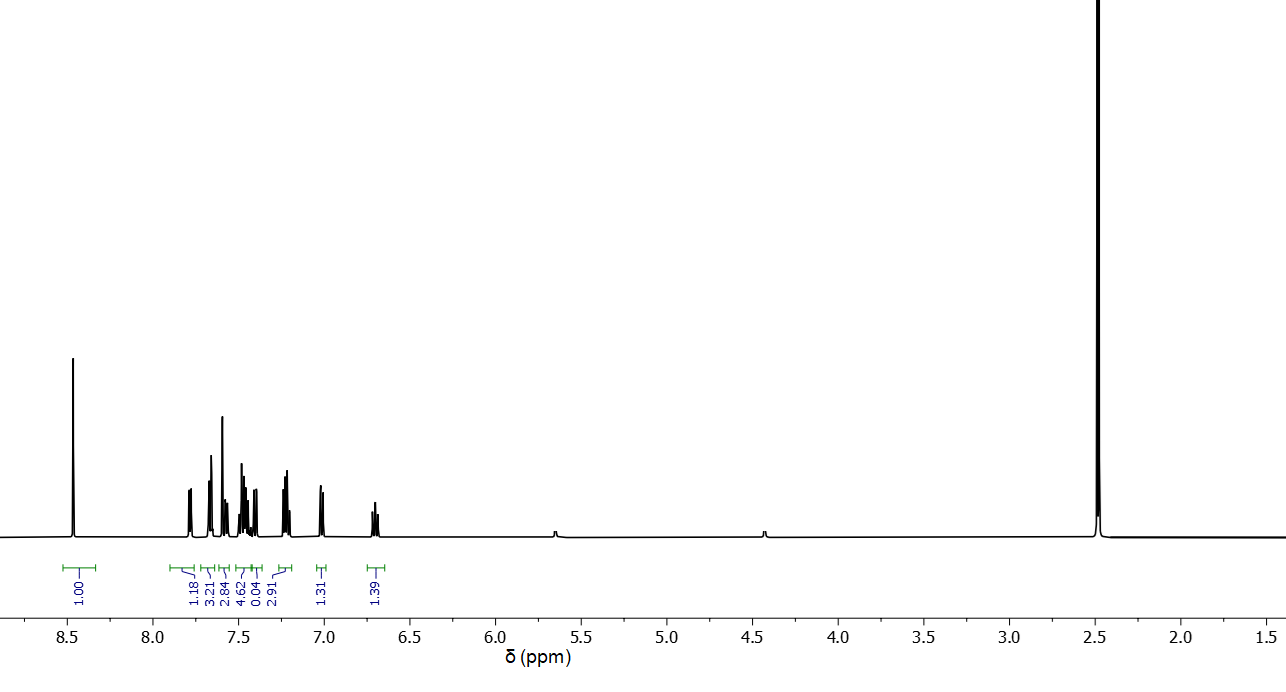


**Fig. S9:** ^1^H NMR of **(**Z)-2-amino-5-((amino(4-(4-hydroxy-3-methoxybenzylidene)-5-oxo-2-phenyl-4,5-dihydro-1H-imidazol-1-yl)methyl)amino)pentanoic acid **(3g)**.


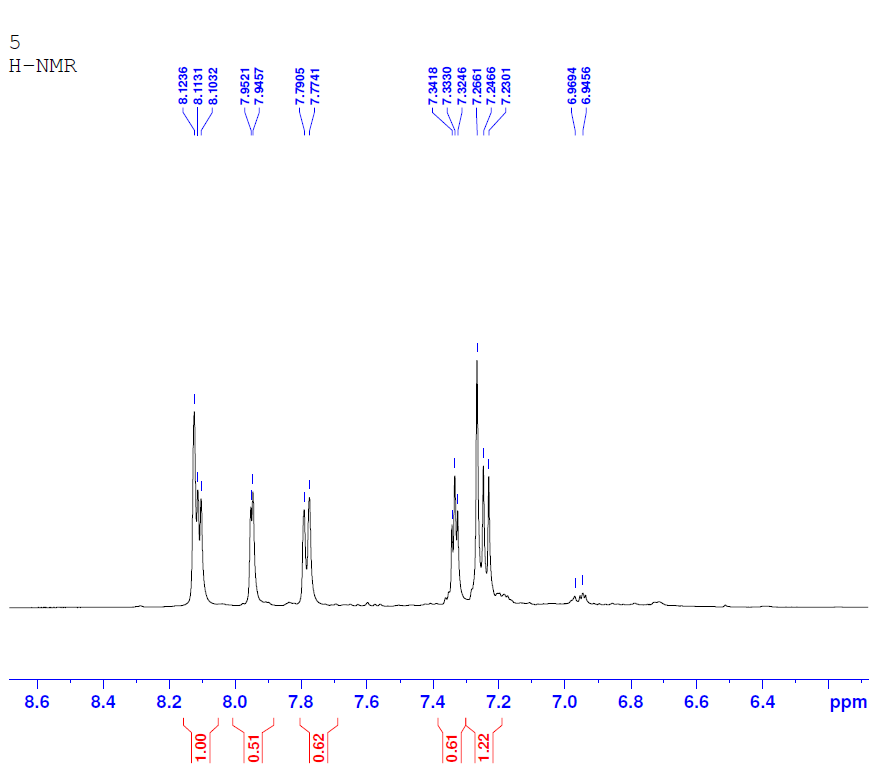


**Fig. S10a:** ^1^H NMR of 2-thiophenecarbonylglycine **(4).**


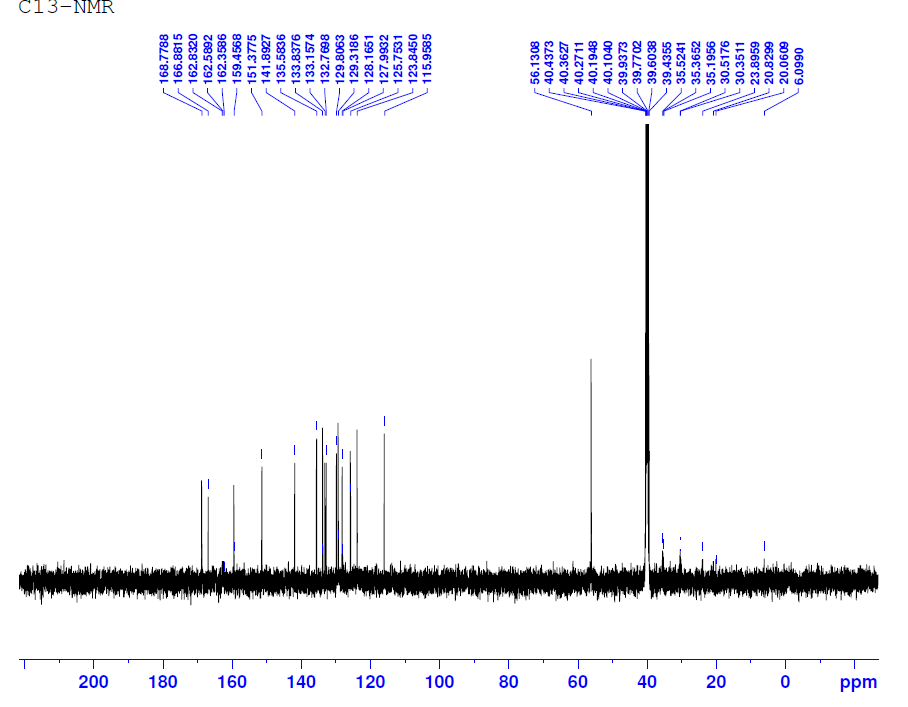


**Fig. S10b:** ^13^C NMR of 2-thiophenecarbonylglycine **(4).**


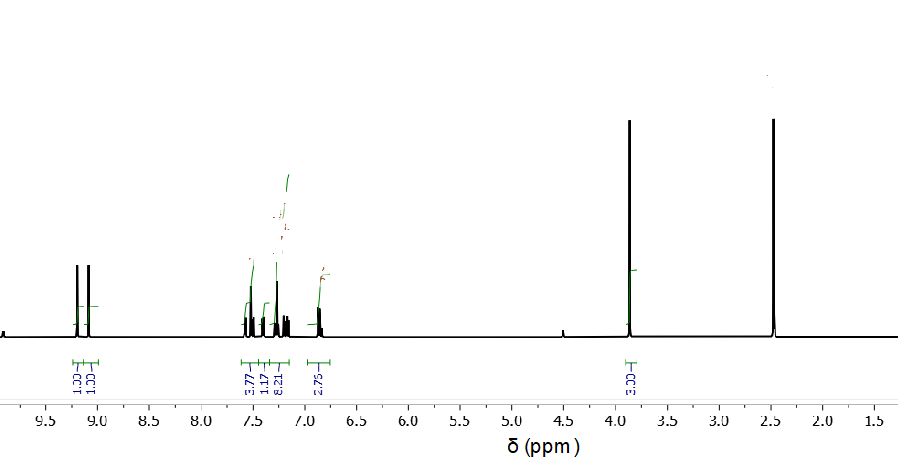


**Fig. S11:** ^1^H NMR of (Z)-3-((2-bromophenyl)amino)-5-(4-hydroxy-3-methoxybenzylidene)-2-(thiophen-2-yl)-3,5-dihydro-4H-imidazol-4-one **(5a)**.


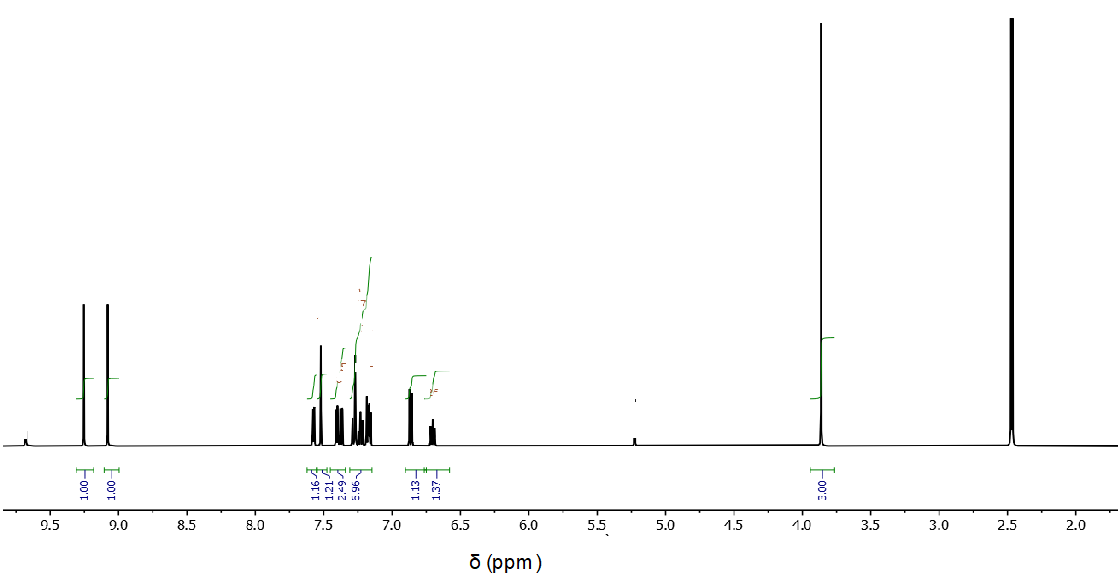


**Fig. S12:** ^1^H NMR of (Z)-3-((2-chlorophenyl)amino)-5-(4-hydroxy-3-methoxybenzylidene)-2-(thiophen-2-yl)-3,5-dihydro-4H-imidazol-4-one **(5b)**.

**
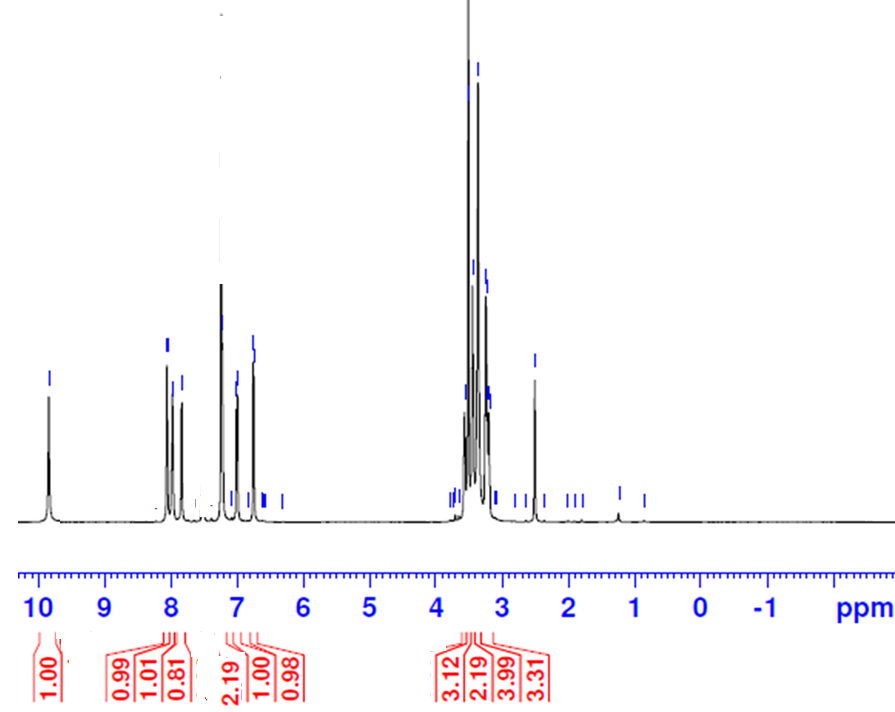
**

**Fig. S13a:** ^1^H NMR of (Z)-5-(4-hydroxy-3-methoxybenzylidene)-3-(2-hydroxyethyl)-2-(thiophen-2-yl)-3,5-dihydro-4H-imidazol-4-one **(5c)**.


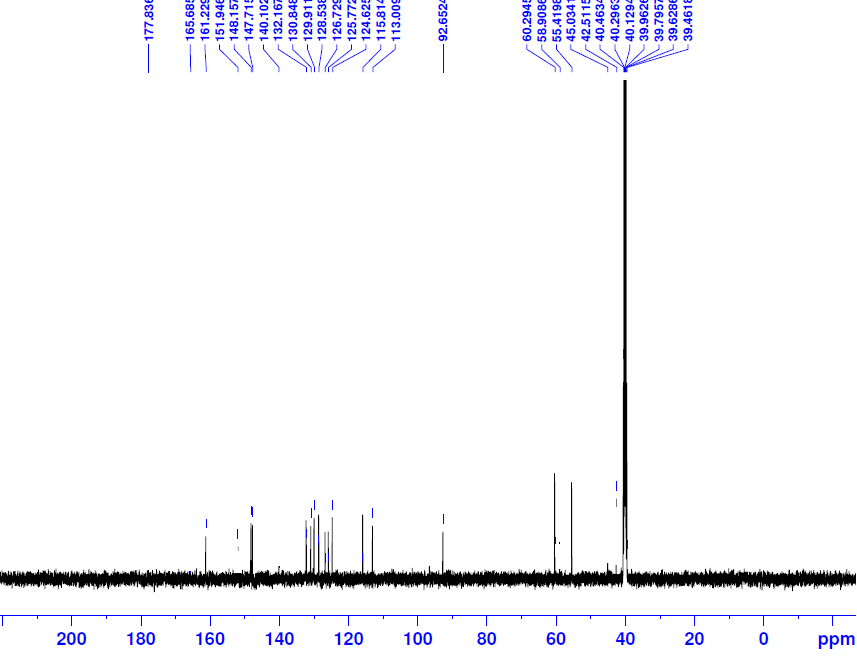


**Fig. S13b:** ^13^C NMR of (Z)-5-(4-hydroxy-3-methoxybenzylidene)-3-(2-hydroxyethyl)-2-(thiophen-2-yl)-3,5-dihydro-4H-imidazol-4-one **(5c)**.


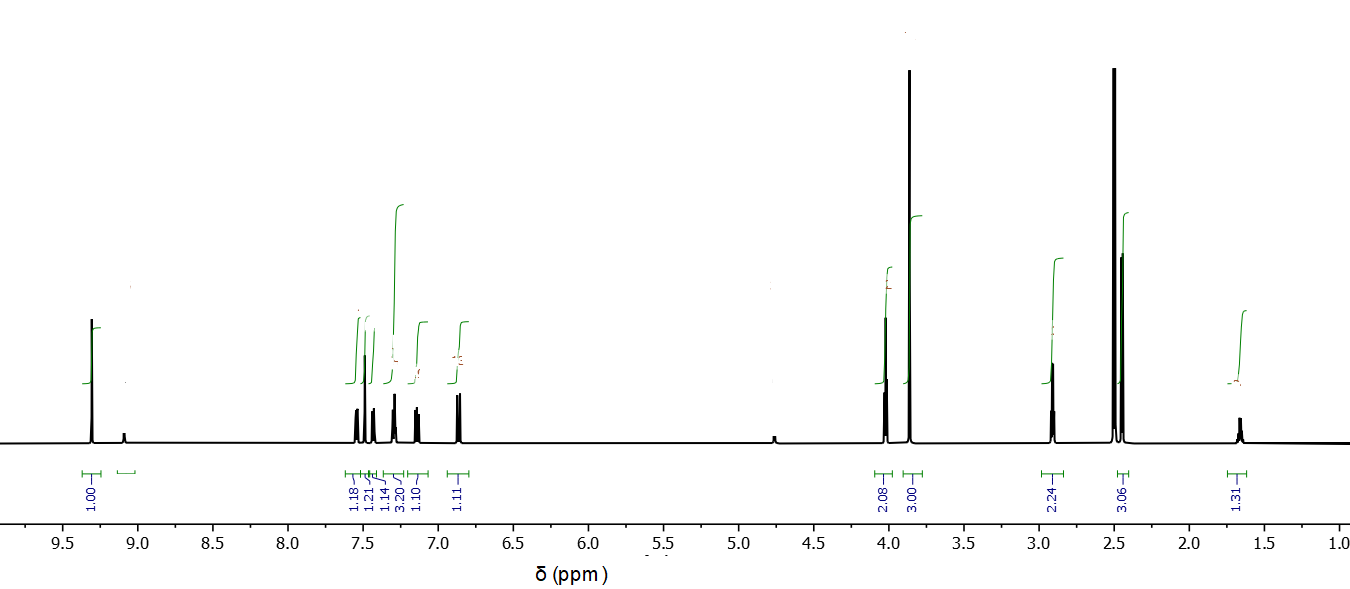


**Fig. S14a:** ^1^H NMR of (Z)-5-(4-hydroxy-3-methoxybenzylidene)-3-(2-(methylamino)ethyl)-2-(thiophen-2-yl)-3,5-dihydro-4H-imidazol-4-one **(5d).**

**
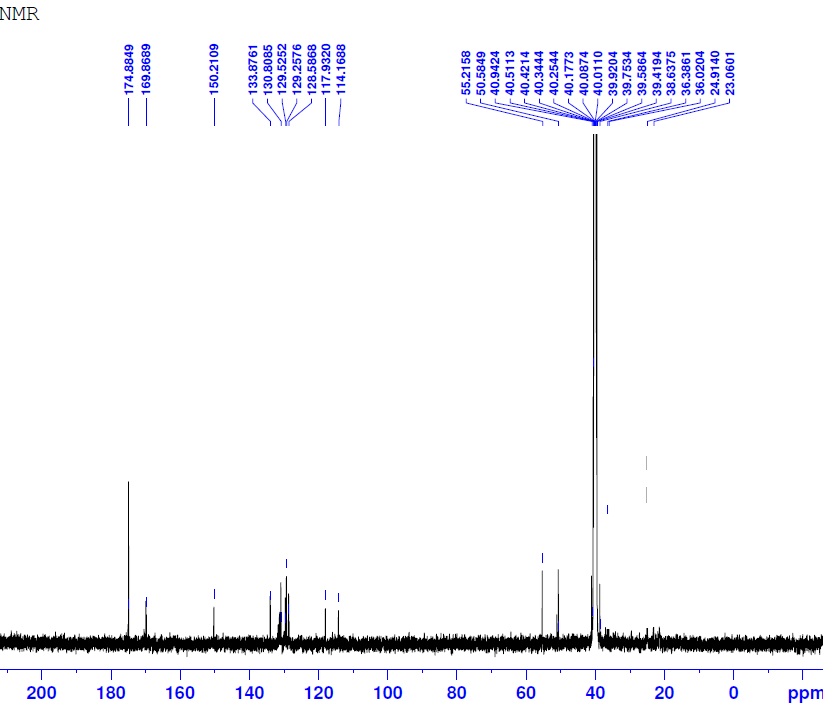
**

Fig S14b. ^1^H NMR of (Z)-5-(4-hydroxy-3-methoxybenzylidene)-3-(2-(methylamino)ethyl)-2-(thiophen-2-yl)-3,5-dihydro-4H-imidazol-4-one **(5d).**


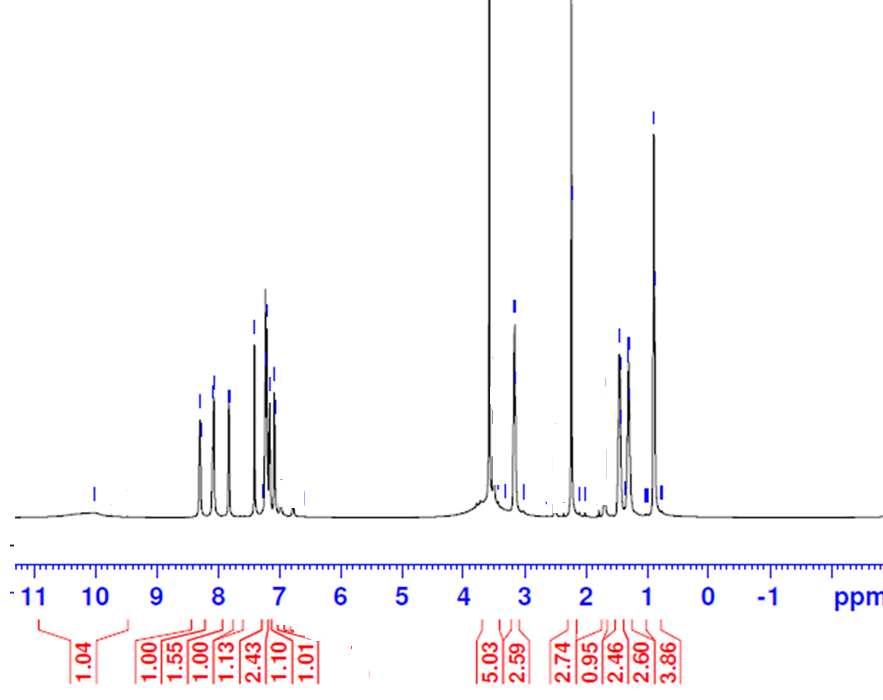


**Fig. S15a:** ^1^H NMR of (Z)-3-butyl-5-(4-hydroxy-3-methoxybenzylidene)-2-(thiophen-2-yl)-3,5-dihydro-4H-imidazol-4-one **(5e).**


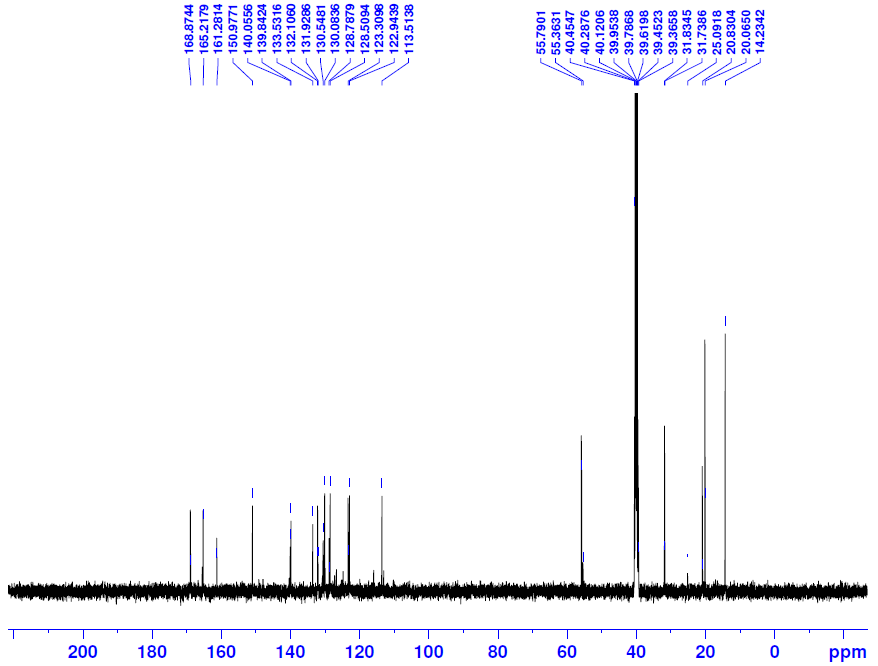


**Fig. S15b:** ^13^C NMR of (Z)-3-butyl-5-(4-hydroxy-3-methoxybenzylidene)-2-(thiophen-2-yl)-3,5-dihydro-4H-imidazol-4-one **(5e).**

**
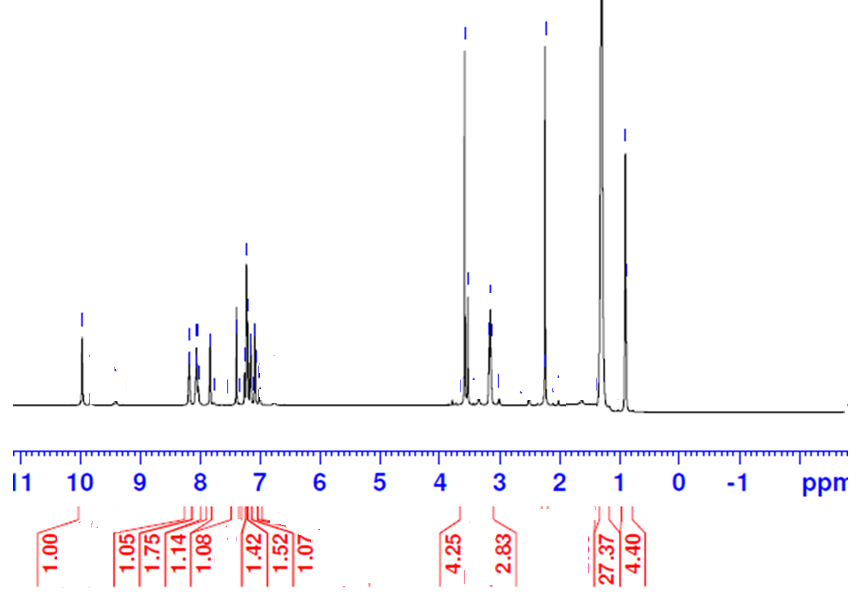
**

**Fig. S16:** ^1^H NMR of (Z)-3-dodecyl-5-(4-hydroxy-3-methoxybenzylidene)-2-(thiophen-2-yl)-3,5-dihydro-4H-imidazol-4-one **(5f).**


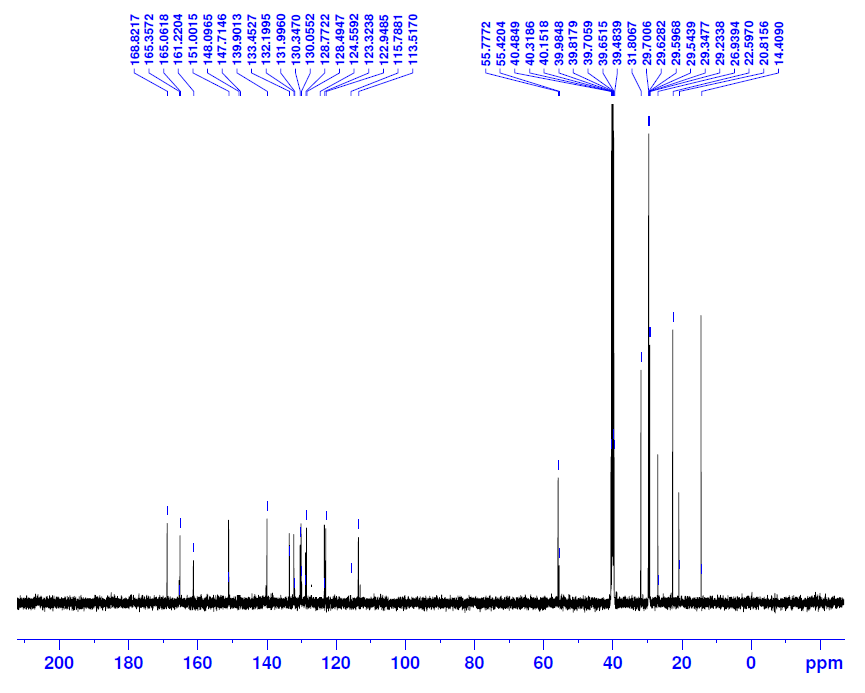


**Fig. S16b:** ^13^C NMR of (Z)-3-dodecyl-5-(4-hydroxy-3-methoxybenzylidene)-2-(thiophen-2-yl)-3,5-dihydro-4H-imidazol-4-one **(5f).**


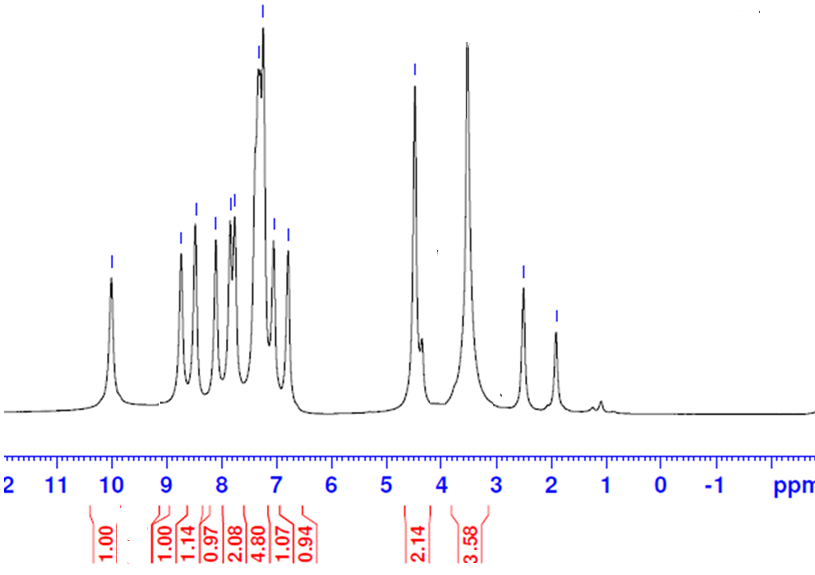


**Fig. S17a:** ^1^H NMR of (Z)-5-(4-hydroxy-3-methoxybenzylidene)-3-(pyridin-3-ylmethyl)-2-(thiophen-2-yl)-3,5-dihydro-4H-imidazol-4-one **(5g).**


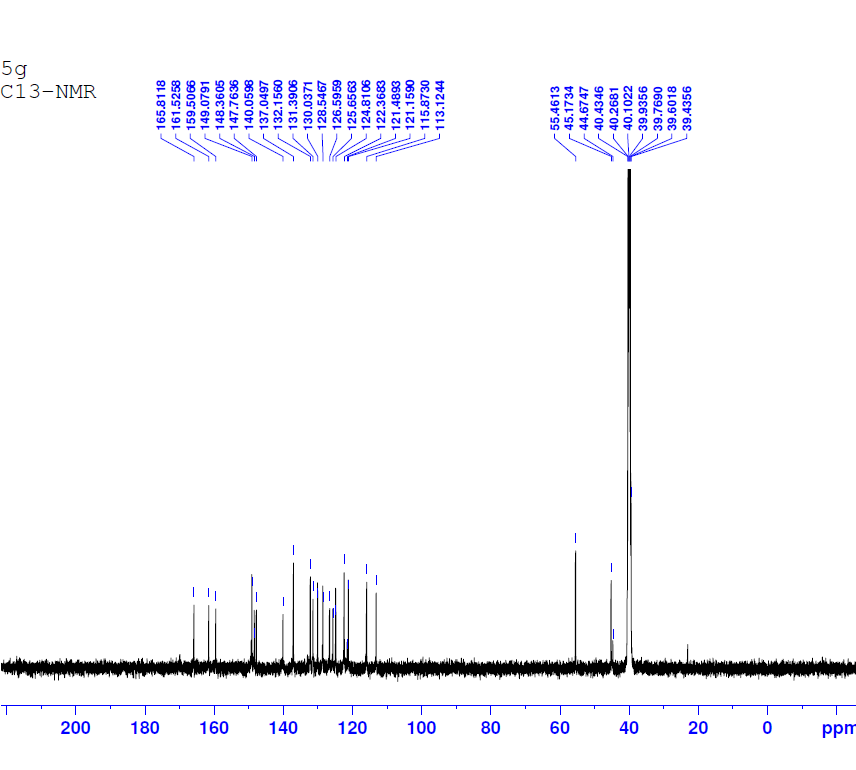


**Fig. S17b:** ^13^C NMR of (Z)-5-(4-hydroxy-3-methoxybenzylidene)-3-(pyridin-3-ylmethyl)-2-(thiophen-2-yl)-3,5-dihydro-4H-imidazol-4-one **(5g).**
